# Beta 2 adrenergic receptor and mu opioid receptor interact to potentiate the aggressiveness of human breast cancer cell by activating the glycogen synthase kinase 3 signaling

**DOI:** 10.1186/s13058-022-01526-y

**Published:** 2022-05-14

**Authors:** Bénédicte Rousseau, Sengottuvelan Murugan, Ajay Palagani, Dipak K. Sarkar

**Affiliations:** Endocrine Research Program, Department of Animal Sciences, Rutgers, The State University of New Jersey, 67 Poultry Farm Lane, New Brunswick, NJ 08901 USA

**Keywords:** Triple-negative breast cancer, Growth and metastasis, Beta-adrenergic receptor, Mu-opioid receptor, GSK3 signaling, Gene knockdown, Pharmacological agents

## Abstract

**Background:**

Opioid and beta-adrenergic receptors are recently shown to cross talk via formation of receptor heterodimers to control the growth and proliferation of breast cancer cells. However, the underlying cell signaling mechanism remained unclear.

**Methods:**

To determine the effect of the interaction of the two systems in breast cancer, we employed triple-negative breast cancer cell lines MDA-MB-231 and MDA-MB-468, CRISPR or chemical inhibition or activation of beta-adrenergic receptors (B2AR) and mu-opioid receptors (MOR) gene, and PCR array technology and studied aggressive tumor phenotype and signaling cascades.

**Results:**

We show here that in triple-negative breast cancer cells, the reduction in expression B2AR and MOR by genetic and pharmacological tools leads to a less aggressive phenotype of triple-negative breast cancer cells in vitro and in animal xenografts. Genomic analysis indicates the glycogen synthase kinase 3 (GSK3) pathway as a possible candidate messenger system involved in B2AR and MOR cross talk. GSK3 inactivation in MDA-MB-231 and MDA-MB-468 cells induced similar phenotypic changes as the inhibition of B2AR and/or MOR, while a GSK3 activation by wortmannin reversed the effects of B2AR and/or MOR knockdown on these cells. GSK3 inactivation also prevents B2AR agonist norepinephrine or MOR agonist DAMGO from affecting MDA-MB-231 and MDA-MB-468 cell proliferation.

**Conclusions:**

These data confirm a role of B2AR and MOR interaction in the control of breast cancer cell growth and identify a possible role of the GSK3 signaling system in mediation of these two receptors’ cross talk. Screening for ligands targeting B2AR and MOR interaction and/or the GSK3 system may help to identify novel drugs for the prevention of triple-negative breast cancer cell growth and metastasis.

**Supplementary Information:**

The online version contains supplementary material available at 10.1186/s13058-022-01526-y.

## Introduction

Breast cancer is the world’s leading cause of cancer mortality in women in the world [1–5]. Research has demonstrated a strong link between chronic stress and a weakened immune system, leaving the body prone to disease, like cancer [6–9]. Stress hormones, such as norepinephrine and epinephrine, acting primarily via β2-adrenergic receptors (B2AR), have been shown to promote tumor growth and differentiation, angiogenesis, migration, and metastasis [10–12]. Additionally, B2AR is highly expressed in different types of cancer, including breast cancer, and is associated with a poor prognosis [13, 14]. Also, recently B2AR blockers have been shown to inhibit metastatic spread and reduce cancer risk [15], providing clinical evidence for the involvement of B2AR in cancer progression. Body stress also affects the production of endogenous opioid peptides, which are critical to control stress homeostasis [16–18]. Opioid receptors are also found to be upregulated in several types of cancer [19, 20]. Administration of morphine, an opioid peptide which binds to μ-opioid receptors (MOR), has been shown to promote tumor growth, tumor apoptosis, angiogenesis, migration, and invasiveness [21–24], while a MOR antagonist, methylnaltrexone, has been shown to possess antiproliferative and antimetastatic properties in lung cancer [25]. Hence, stress promotion of cancer might involve both B2AR and MOR.

Both MOR and B2AR are G-protein-coupled receptors (GPCRs) and are closely related receptor systems and coexist in many cells, including breast tumor cells [10, 11, 26]. Within the breast cancer cell lines, these receptors are present in both triple-negative breast cancer (TNBC) lines like MDA-MB-231 and MDA-MB-468 cells that represent the basal B and basal A subtypes of breast cancer, and non-TNBC lines like MCF-7 and T47D cells that represent the luminal A subtype of breast cancer [27–29]. GPCRs are known to heterodimerize with closely related members, resulting in the modulation of their functions [12, 13, 15]. Ligand binding studies have shown that opioidergic and adrenergic agents can bind to their complementary receptors and enhance the activity of other compound types through an allosteric mechanism [20]. Physical interaction of B2AR and MOR has also been reported by co-immunoprecipitation studies in breast cancer cells [29], in CHO cells exogenously expressing both receptors [30], and in neuronal cells [31]. These data support the notion that opioidergic and adrenergic systems may interact to modulate cellular processes. However, opioidergic and adrenergic systems interaction in breast cancer cells remain unclear. We recently have shown that propranolol (PRO), a beta blocker and naltrexone (NTX), an opiate antagonist, when combined, produce marked inhibitory effects on breast tumor growth and tumor mass while improving the survival rate [29]. These antitumor effects resulted from a reduction in tumor cell proliferation, the induction of cellular apoptosis, and the prevention of epithelial–mesenchymal transition in the tumor. However, the underlying cell signaling mechanism remained unclear. We demonstrate in this study that B2AR and MOR interact to control triple-negative breast cancer (TNBC) cell growth and progression via activation of the GSK3 signaling system.

## Materials and methods

### Mice

The animal care and treatment were performed in accordance with institutional guidelines, and protocols were approved by the Rutgers Institutional Animal Care and Facilities Committee (PROTO 999900286) and complied with National Institutes of Health policy.

### MDA-MB-231 and MDA-MB-468 cell line

Breast cancer cell line MDA-MB-231 and MDA-MB-468 was obtained from the ATCC® (reference HTB-26™ and HTB-132™; Gaithersburg, MD). The cell lines were cultivated in DMEM high glucose medium (Gibco; Gaithersburg, MD) with a final concentration of 10% fetal bovine serum (FBS) (Gibco) and 1% antibiotic–antimycotic (Gibco). Cells were trypsinized every 3 to 4 days, never exceeding 10 passages in all experiments, and maintained at 37 °C and 5% CO_2_.

### CRISPR-Cas9 knockdown

The specific design of forward and reverse primers used to generate the gRNA by in vitro transcription (IVT) was done with the help of the Invitrogen™ GeneArt™ CRISPR Search and Design tool (http://www.thermofisher.com/crisprdesign). The CRISPR design tool identifies the top six CRISPR target sequences. Among them, three paired primers were selected for each target gene. (The different CRISPR sequences used are listed (Additional file 1: Table S1.) The full-length gRNA DNA template was generated by PCR using the previously forward and reverse overlapping oligonucleotides. These primers contain the target DNA sequence (CRISPR sequence) and the Tracr Fragment and T7 Primer Mix. They play the dual role of primer and template. The gRNA sequence has been generated by in vitro transcription using the TranscriptAid™ Enzyme Mix and then it has been purified using GeneJet™ RNA Purification Micro Columns. All the procedures were done using the GeneArt™ Precision gRNA Synthesis Kit (Invitrogen). Before the day of the transfection, MDA-MB-231 and MDA-MB-468 cells were trypsinized and seeded into a 24-well plate (3.2 × 10^5^ cells/mL). After changing media, the transfection was done using Opti-MEM I reduced serum medium, GeneArt™ CRISPR Nuclease mRNA (Invitrogen, Thermo Fisher Scientific; Princeton, NJ), and Lipofectamine® CRISPRMax™ Reagent (Invitrogen). Three gRNAs were used to target each gene (Additional file 1: Table S1). Two controls (one media only control another Lipofectamine only control) were used. Cells were maintained for 48 h at 37 °C and 5% CO_2._

### Subcutaneous Xenograft experiments

T-cell-deficient, athymic nude (Crl: NIH-Foxn1rnu) female rats aged 21–28 days old were purchased from Charles River (Portage, MI, USA) and maintained in a pathogen-free condition with a 12-h light/dark cycle at our institute’s animal research facility. Animal care and treatment were performed in accordance with institutional guidelines, and protocols were approved by the Rutgers Institutional Animal Care and Facilities Committee and complied with National Institutes of Health policy.

MDA-MB 231 cells, at a final concentration of 1 × 10^7^ cells/rat in 200-µl of PBS-50% Matrigel (BD Biosciences; San Jose, CA) mixture, were injected subcutaneously (SC) into the right flank of the athymic nude female rats (Crl: NIH-Foxn1rnu; Charles River; Portage, MI, USA). After tumors reached a diameter of approximately 50 mm^3^, the animals were randomly assigned to different treatment groups and injected s.c. daily with saline (control), NTX (10 mg/kg Sigma-Aldrich, St. Louis, MO, USA), PRO (10 mg/kg, Sigma-Aldrich, St. Louis, MO, USA), or in combination for 3 weeks. Tumor shrinkage/growth was measured in animals daily and animal weights were measured every other day. Animals were euthanized when the tumor reached 5000 mm^3^. Three dimensions of each tumor were measured using electronic calipers, and tumor volumes were calculated by the formula L x W^2^/2. The mean ± SEM of tumor volume was calculated weekly for each experimental group and presented. After the animals were sacrificed, the tumors were excised and were snap-frozen by immersion in liquid nitrogen and stored at − 80 °C until further use.

### Cell growth assay

Experiments were performed in 96‐well plates over 72 h (1.5 × 10^5^ cells/mL). Cell growth was estimated at 0, 24, 48, and 72 h by a colorimetric assay based on the conversion of tetrazolium dye (MTT) to a blue formazan product by live mitochondria. Optical density at 595 nm corresponding to solubilized formazan was read for each well on a Multiskan FC (Thermo Scientific Branchburg, NJ, USA). Data are presented as percent of control at 72 h.

### Migration assay

Transwell (8 µm pore size insert) migration assay was performed in 24-well plates. Lower compartments contained DMEM (Gibco, Thermo Scientific, Branchburg, NJ, USA) with 0.5% FBS (Gibco, Thermo Scientific, Branchburg, NJ, USA) and 40 µg/mL collagen (Sigma-Aldrich, St. Louis, MO, USA). Upper compartments contained the cells in DMEM with 0.5% (5 × 10^5^ cells/mL). Cells were incubated in the Transwell plate at 37 °C and 5% CO_2_ for 3 h. Then they were fixed on the lower side of the insert filter using 5% glutaraldehyde for 10 min and stained with crystal violet for 20 min. Cell density was measured using Image J analysis software (National Institutes of Health).

### Colony formation assay

Experiments were performed in 6-well plates (10^3^ cells/mL). Cells were incubated for 14 days at 37 °C and 5% CO2, with fresh media added every 3 days. After carefully washing with 1X phosphate-buffered saline (PBS), colonies were stained with crystal violet for 15 min. Cell density was measured using Image J analysis software (National Institutes of Health).

### Treatments

Cells were treated by SB-216763 (Sigma-Aldrich; Allentown, PA), wortmannin (Sigma-Aldrich, St. Louis, MO, USA), epinephrine ((Sigma-Aldrich, St. Louis, MO, USA)), and [D-Ala2, N-MePhe4, Gly-ol]-enkephalin (DAMGO) ((Sigma-Aldrich, St. Louis, MO, USA)). Experiments were performed in 96-well or 6-well plates (5 × 10^5^ cells/mL).

### Cancer stem cell gene qPCR array

The human cancer stem cell RT^2^ Profiler PCR array (Qiagen; Frederick, MD) profiles the expression of 84 genes linked to cancer stem cells (CSCs). The genes profiled with this array include CSC molecular markers and genes regulating CSC proliferation, self-renewal, or pluripotency and also those involved in CSC asymmetric cell division, migration, and metastasis. They are all listed in Additional file 1: Table S2. Synthesis of cDNA was done using the RT^2^ First Strand Kit (Qiagen; Frederick, MD) with 0.5 µg total RNA. Real-time PCR of the 96-well PCR array was performed using RT^2^ SYBR Green Mastermix on an ABI 7500 (Applied Biosystems™; Wilmington, DL). A set of controls present in the array enabled analysis using the ΔΔC_T_ method using the Data Analysis Center (Qiagen; Frederick, MD). Fold regulation values were then used to design a heatmap with gplot package (heatmap2) in R Studio and to analyze canonical pathways with Ingenuity Pathway Analysis (IPA, Qiagen; Frederick, MD).

### qPCR

Real-time RT-PCR was carried out in 384-well plates in a final volume of 10 µL with 1:30 dilution cDNA (1 µg/µL) in *Power* SYBR® Green PCR Master Mix (Applied Biosystems™) using the comparative C_T_ (ΔΔC_T_) standard run on a the QuantStudio™ 7 Flex Real-Time PCR System (Applied Biosystems™, Wilmington, DL). The primers were designed using Primer 3 (http://bioinfo.ut.ee/primer3/). Analysis of the results was done using QuantStudio™ Real-Time PCR (Applied Biosystems™, Wilmington, DL) software. The primers used are listed in Additional file 1: Table S3; four genes were used as housekeeping control genes: GAPDH, ATCB, B2M, and HPRT1.

### Western blot

For immunoblotting, about 50 µg of total protein from MDA-MB-231 or MDA-MB-468 cells were run in 12% SDS PAGE and transferred to PVDF membranes (GE Health Care, Piscataway, NY, USA) at 75 V for 2 h at 4 °C. The membranes were blocked in 5% non-fat dry milk-TBS-0.1% Tween 20 (TBST) at room temperature for 2 h and then incubated with primary antibody in the same buffer at 4 °C overnight. The following primary antibodies were used: antibodies against B2AR (1:1000, Abcam, Branford, CT, RRID: AB_2747383), MOR (1:1000, Cell Signaling, Danvers, MA, RRID: AB_177512) GSK3α (1:1000, Cell Signaling, Danvers, MA, RRID: RRID:AB_659836), GSK3β (1:1000, Cell Signaling, Danvers, MA, RRID: RRID:AB_10998934), and β-actin (1:10,000, Abcam, Branford, CT, RRID:AB_2737344). The membranes were washed for 10 min 6 times in TBST and then incubated with corresponding peroxidase conjugated secondary antibody (1:5000, Vector Labs, Burlingame, CA) at room temperature for 1 h. The membranes were washed in TBST for 10 min 6 times and incubated with ECL reagent (Thermo Fisher Scientific, and were developed on the film by autoradiography. The protein band intensities were determined by Image J analysis software (National Institutes of Health, Bethesda, MD) and normalized with corresponding β-actin band intensity.

### Statistical analysis

Prism 7.0 (GraphPad Software) was used for statistical analysis. The data were expressed as means ± SEM values of three independent experiments. Significance of difference was analyzed using one-way analysis of variance (ANOVA) with post hoc analysis using the Newman–Keuls posttest. For all analyses, values of *p* < 0.05 were considered significant.

## Results

### Inhibition of the expression of B2AR and MOR receptors alone or together reduces cell proliferation, cell migration, and colony formation of MDA-MB-231 and MDA-MB-468 cells

We have recently shown that B2AR and MOR interact to reduce the growth and proliferation of three human breast cancer cells [29]. We first confirmed the interactive actions of B2AR and MOR on MDA-MB-231 and MDA-MB-468 cell functions. Two technological approaches were used to determine the role of B2AR and MOR in the regulation of growth, migration, and invasion properties of MDA-MB-231 and MDA-MB-468 cells: one involving CRISPR technology to knock down B2AR and MOR receptors and another involving the pharmacological blockade of these two receptors with the use of PRO and NTX.

**Fig. 1:**
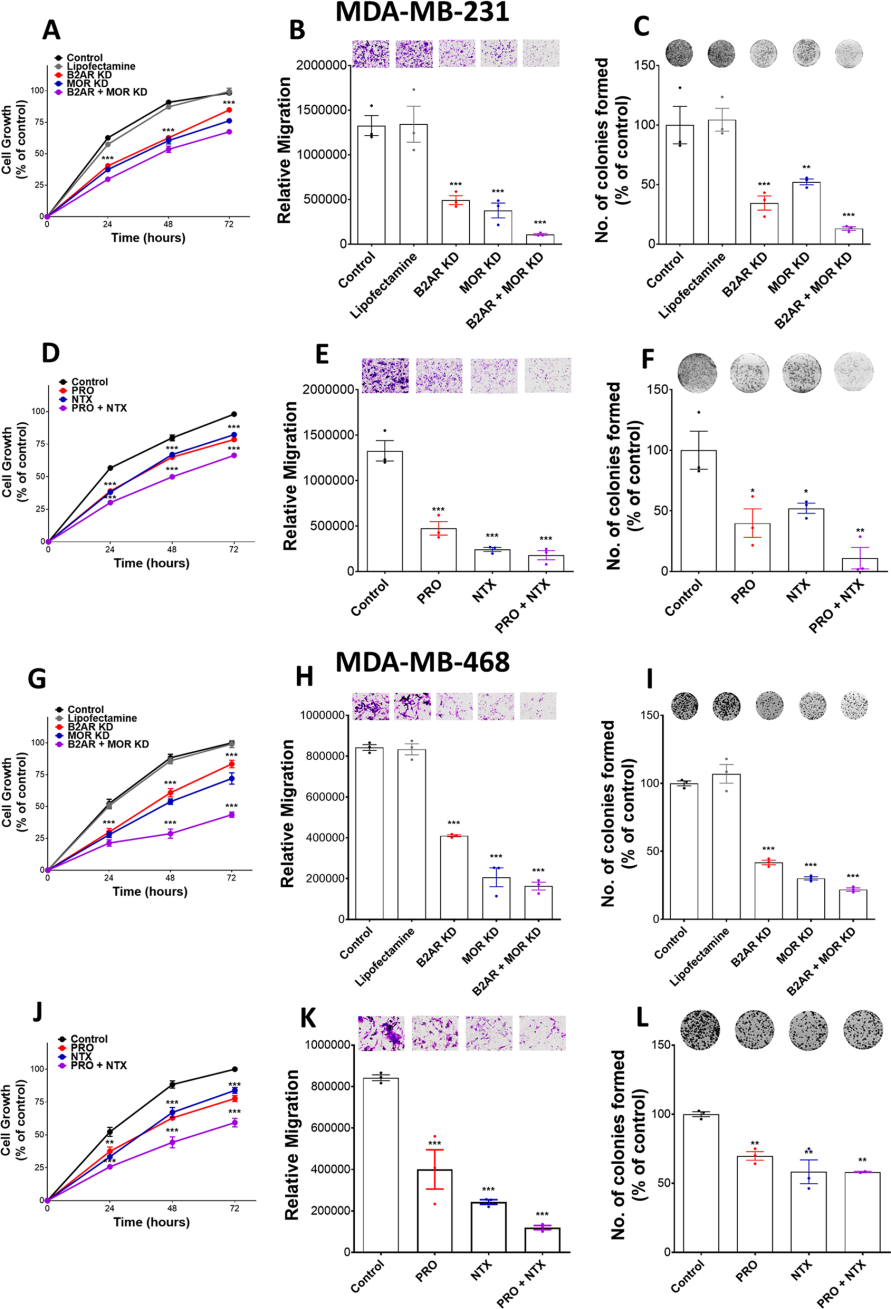
B2AR and MOR knockdown reduces cell proliferation, cell migration, and colony formation in breast cancer cells. B2AR and/or MOR gene expression suppression was done by CRISPR technology or pharmacological blockade of PRO (100 µM), NTX (100 µM), or PRO + NTX in MDA-MB-231 cells. **A**, **D**, **G**, **J** Cell proliferation was evaluated at 0, 24, 48 and 72 h using MTT assay. **B**, **E**, **H**, **K** Cell migration capacity was evaluated using the Transwell cell migration assay. Cells were stained with crystal violet after 3 h. Representative images are shown on the top. Images were analyzed using ImageJ. **C**, **F**, **I**, **L** Colony formation by cells was evaluated for 2 weeks. After 14 days, colonies were stained using crystal violet. Representative images are shown on the top. Images were analyzed using ImageJ. Data are mean ± SEM values obtained from three independent experiments. *p < 0.05, **p < 0.01, ***p < 0.001

In the CRISPR knockdown approach, 3 gRNAs were used for each gene and no treatment control and lipofectamine treatment was used as CRISPR negative control. Western blot assays were conducted to verify the effectiveness of the gene knockdown by CRISPR (Additional file 1: Fig. S1). Cell growth response to various treatments was evaluated at 24-h intervals for 72 h using MTT assay. We show that CRISPR knockdown of B2AR and MOR alone or in combination inhibited the growth of MDA-MB-231 (Fig. 1A) and MDA-MB-468 cells (Fig. 1G) at all time points as compared to those in control groups. The highest cell growth inhibition was seen in the B2AR and MOR combined knockdown group at all time points. The capacity of cells to migrate following B2AR, MOR, and B2AR + MOR knockdown was also evaluated using a Transwell membrane and collagen I as a chemoattractant molecule for 3 h (Fig. 1B, H, Additional file 1: Fig. S2A, B). The loss of expression of MOR or B2AR significantly reduced the capacity of MDA-MB-231 and MDA-MB-468 cells to migrate as compared to the controls. The lowest cell migration was seen when both receptors were inhibited. Finally, MOR and B2AR knockdown cells also show a lesser ability to form colonies compared to controls, with the double knockdown cells being the most impacted compared to the single knockdown (Fig. 1C, I, Additional file 1: Fig. S2C and S2D). These results suggest that both B2AR and MOR act individually or interactively to control MDA-MB-231 and MDA-MB-468 cell growth, migration, and invasion functions.

The effects of the treatment of 100 µM (IC50 dose; [29]) of PRO or NTX alone or in combination at 24-h intervals for 72 h on cell growth, colony formation, migration, and invasion were determined. Treatment with PRO and NTX alone or in combination significantly reduced cell growth response as determined by MTT assay (Fig. 1D, J). Interestingly, maximal growth inhibition was observed when NTX and PRO were combined, while only moderate inhibition was achieved by PRO and NTX when given alone as compared to the control group. The effects of these drugs on the capacity of cells to migrate were evaluated using a Transwell membrane and collagen I as a chemoattractant molecule (Fig. 1E, K, Additional file 1: Fig. S2A and S2B). PRO or NTX treatment alone decreased the migration of the MDA-MB-231 and MDA-MB-468 cells as compared to the controls. Combination treatment of these two drugs markedly decreased the capacity of cells to migrate as compared to the control. Next, the effectiveness of a 100 µM dose of PRO and NTX on colony formation of MDA-MB-231 and MDA-MB-468 was tested (Fig. 1F, L, Additional file 1: Fig. S2C and S2D). The results show that PRO and NTX moderately decreased the number of colonies alone. The combination of PRO + NTX produced the maximum inhibition of colony formation in these cells.

### B2AR and MOR interaction may involve GSK3 signaling

**Fig. 2:**
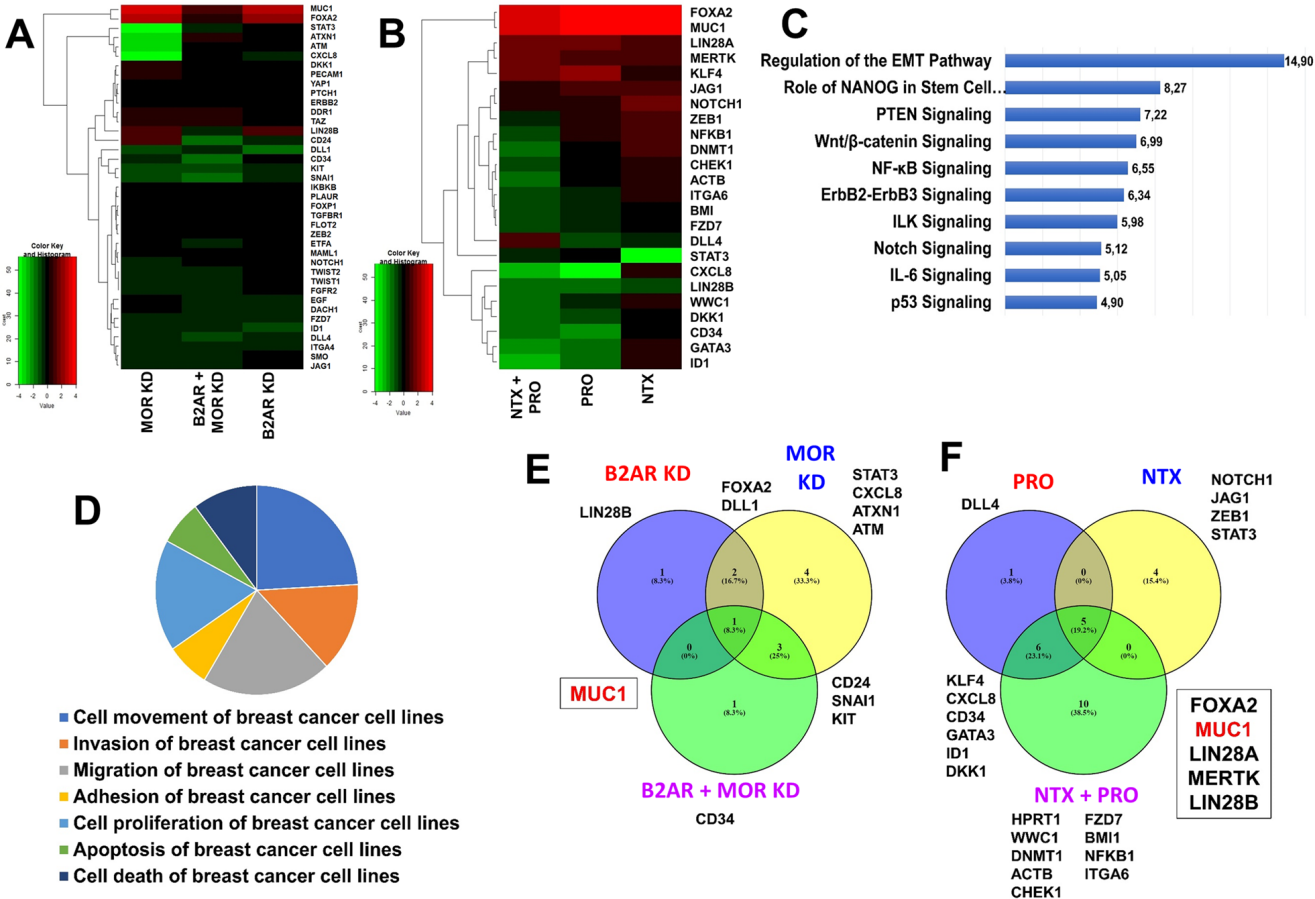
Gene expression profile changes following B2AR and MOR knockdown. Changes in gene expression profiles following knockdown of B2AR with or without MOR in MDA-MB-231 cells were measured using the cancer stem cell PCR array system. **A**, **B** Heatmap analysis of the data in cells following CRISPR knockdown or pharmacological knockdown of B2AR with or without MOR are shown in panels A and B, respectively. Genes with high expression are shown in red, while genes with low expression are shown in green. **C** Canonical pathways that were significantly affected by B2AR and MOR knockdown are listed as high-to-low fold differences. **D** Gene Ontology classification of genes based on biological process shown. **E**, **F** Venn View of significant gene overlap in each inhibited condition: knockdown (**E**) and chemicals (**F**). Genes expressed in the different groups are listed (https://bioinfogp.cnb.csic.es/tools/venny/). N = 3

The inhibition of B2AR or/and MOR receptors induced modifications of the MDA-MB-231 and MDA-MB-468 phenotype with a decrease in cell proliferation, cell migration, and colony formation; all three important functions are used by the cancer cell to maintain its growth and progression [32]. Therefore, we determined if there was a common signaling mechanism these two receptors used to promote this cancer cell growth and progression. For this, we employed a cancer stem cell PCR array system which detects modulation of transcript abundance of sets of genes involved in regulation of cancer cell growth and progression (List of genes in Additional file 1: Table S2). We also employed both pharmacological and genetic approaches to knock down B2AR and MOR functions in MDA-MB-231 cells. Changes of the gene transcription profiles following knockdown of B2AR with or without MOR are shown in Fig. 2. Between 39 and 24 transcripts were found to be differentially expressed as shown in the heatmap for the gene knockdown (Fig. 2A) and the pharmacological blockade conditions (Fig. 2B). Upregulated genes are presented in red and downregulated genes are in green in the subset figures. Fold change values of the gene transcripts were then analyzed by Gene Ontology tools for different gene clusters and by IPA (Ingenuity Pathway Analysis) for canonical pathways. Significant changes were found in gene clusters involved in biological process such as cell movement, invasion, migration, adhesion, cell proliferation, apoptosis, and cell death of breast cancer cell lines (Fig. 2C) and the cell signaling pathways involved in these biological processes (Fig. 2D). The Venn diagrams, performed using the online Venny tool (https://bioinfogp.cnb.csic.es/tools/venny/), of the genes that were expressed in the three different conditions (B2AR inhibition, MOR inhibition, and both B2AR and MOR inhibition) identified several groups of genes modulated under a specific treatment condition, but MUC1 expression is only modulated in all three conditions in both gene knockdown (Fig. 2E) and pharmacological blockade studies (Fig. 2F).

**Fig. 3:**
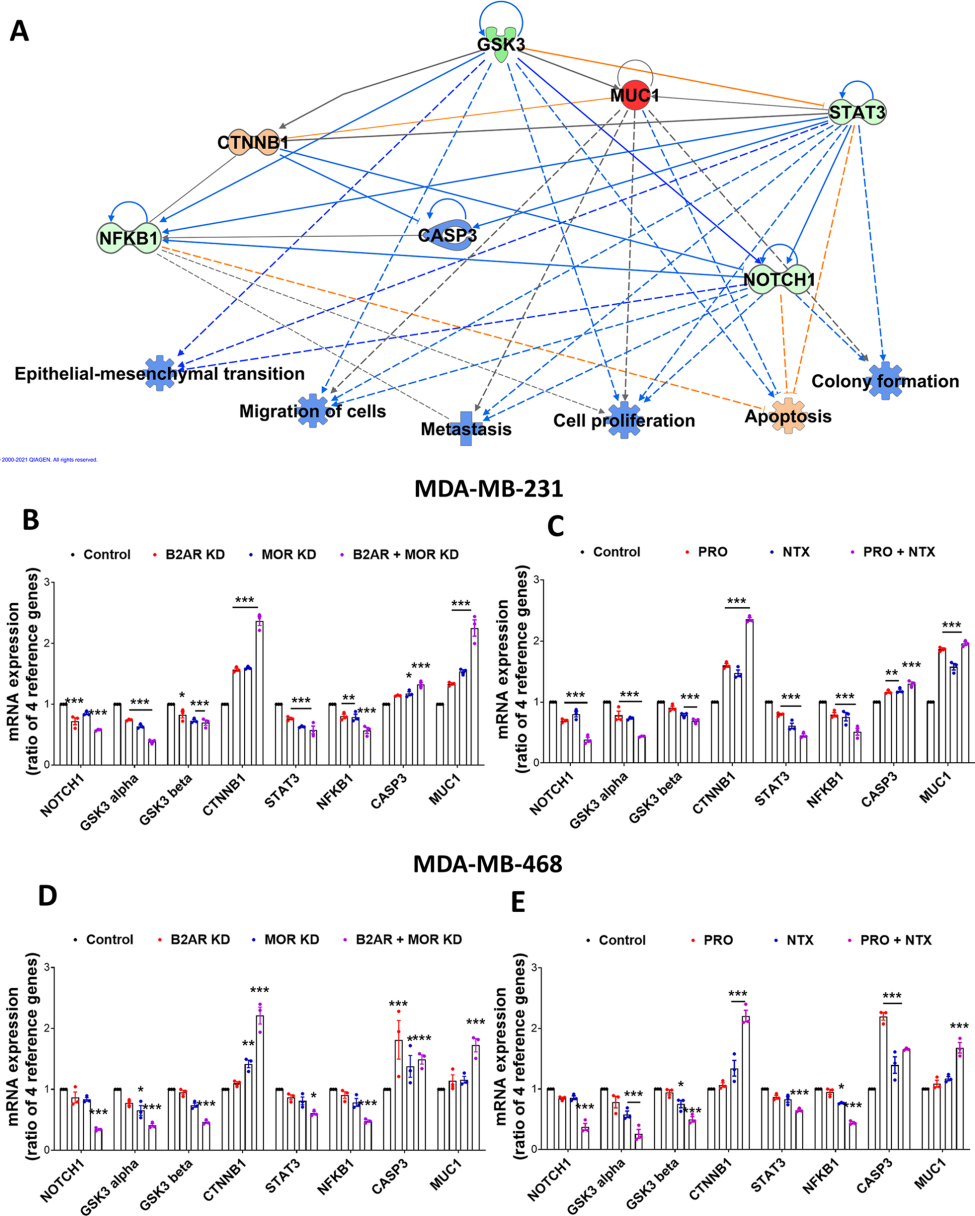
Glycogen synthesis kinase 3 (GSK3) signaling is a candidate mediator of B2AR and MOR interaction. **A** The gene expression changes as determined by the CSC PCR array following B2AR and MOR double knockdown were analyzed by Ingenuity Pathway Analysis (IPA) using a threshold [log2(fold-change)] > 1 or < − 1 as compared to control values. Genes that are part of the PCR array dataset and are upregulated are represented in red, and those that are downregulated are represented in green. The Molecule Activity Predictor (MAP) tool was then used to predict activation or inactivation of downstream genes and principal functions of the cell. Orange indicates predicted activation and blue indicates predicted inactivation. The lines correspond to the relationship(s) between different genes or between gene(s) and cell function(s). Orange lines predict activation of the gene–gene or gene-function relation and blue lines predict inactivation of the gene–gene or gene-function relation. Gray lines indicate no significant prediction. **B**, **C** Validation by RT-PCR measurements of genes relevant to GSK3 signaling and predicted from IPA analysis in MDA-MB-231 and MDA-MB-468 cells with B2AR, MOR, or both B2AR and MOR knockdown by CRISPR (**B**, **D**) or by pharmacological agents PRO, NTX, or both PRO and NTX (**C**, **E**). Normalization of each gene in RT-PCR assay was done using four housekeeping genes (GAPDH, ACTB, B2M, and HPRT1). Data are mean ± SEM (*n* = 3). *p < 0.05, **p < 0.01, ***p < 0.001

Further analysis of PCR array gene data was performed using the IPA My Pathway tool, Molecular Activity Predictor (MAP), in order to identify the predictive pathways altered following genetic or pharmacological inhibition of B2AR with or without MOR. This analysis identified upregulation of the MUC1 gene [33]. In addition, it identified downregulation of GSK3 signaling, which is known to negatively influence MUC1 and CTNNB1 expression [34] and positively affect STAT3, NFKB1, and NOTCH1 expression to control cell proliferation, apoptosis, colony formation, epithelial-mesenchymal transition, migration, and metastasis (Fig. 3A; [35]). Verification of the predicted gene expression changes was conducted using qPCR determination of GSK3 pathway related genes in MDA-MB-231 and MDA-MB-468 cells with knockdown of B2AR, MOR, or B2AR and MOR (achieved by both genetic and pharmacological approaches). As predicted, we found increased levels of MUC1 and CTNNB1 expression and decreased levels of GSK3, NOTCH1, STAT3, and NKB1 expression (Fig. 3B–E). These data led us to hypothesize that activation of B2AR and MOR receptors increases GSK3 signaling to control growth and progression of breast cancer cells.

**Fig. 4:**
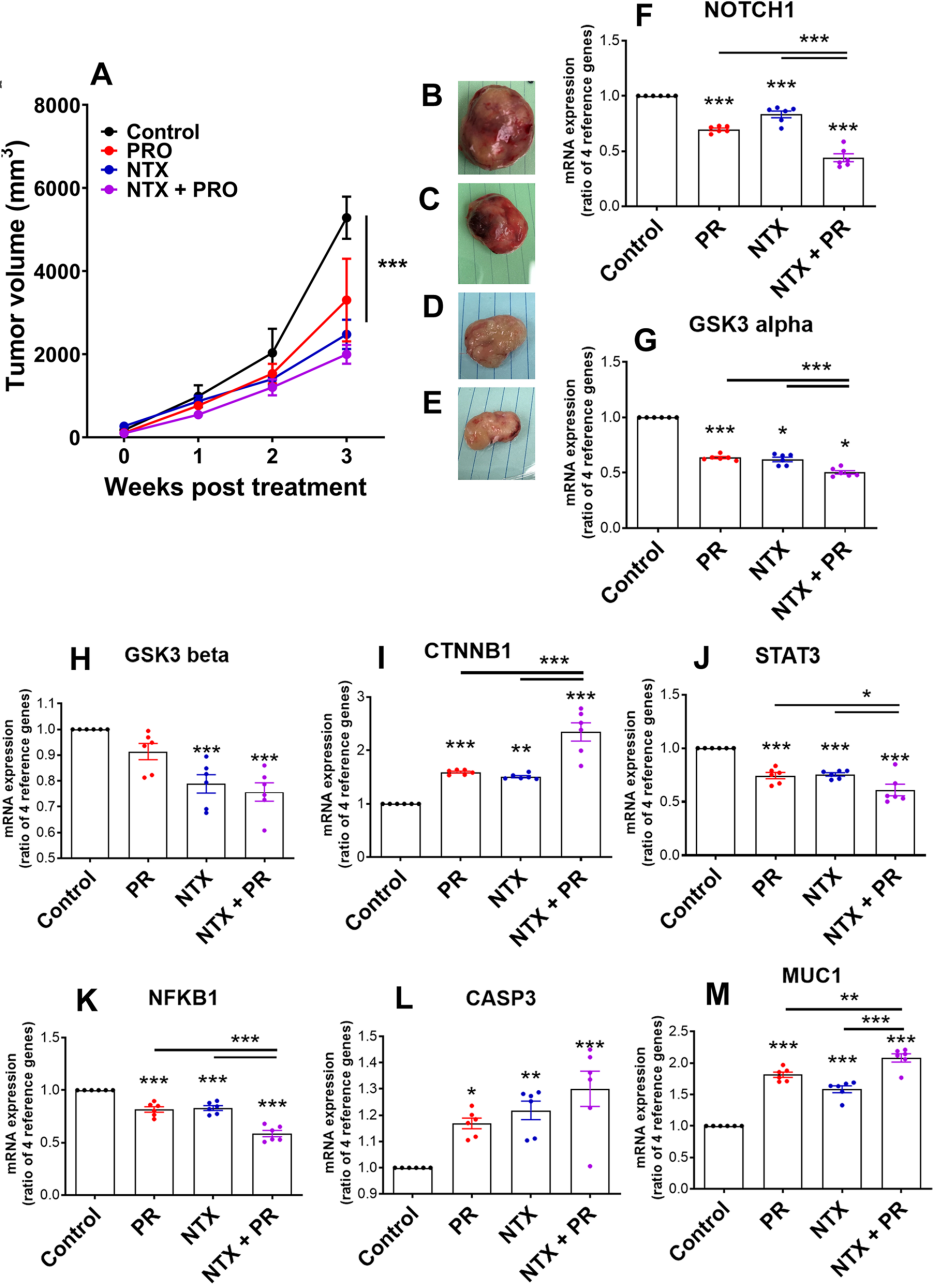
Glycogen synthesis kinase 3 (GSK3) signaling is a candidate mediator of B2AR and MOR interaction: in vivo studies using MDA-MB-231-cell-derived xenografts in nude rats. B2AR and/or MOR gene expression suppression was done by pharmacological blockade of PRO (10 mg/kg body weight)), NTX (10 mg/kg body weight), or PRO + NTX in MDA-MB-231-cells-derived xenografts. **A** Changes in the tumor volume following drug treatments. Values are mean ± SEM (*n* = 6/group). Comparison of tumor volume between groups was done by two-way ANOVA with Bonferroni's multiple comparisons test. ***p < 0.001 vs. control. **B**–**E** Changes in gross morphology and size differences of representative excised xenograft tumors from the control (**B**), PRO (**C**), NTX (**D**), and NTX + PRO (**E**) at 21 days after treatments. **F**–**M** RT-PCR measurements of genes relevant to GSK3 signaling and predicted from IPA analysis in MDA-MB-231 cells with B2AR, MOR, or both B2AR and MOR knockdown by pharmacological agents PRO, NTX, or both PRO and NTX. Normalization of each gene in the RT-PCR assay was done using four housekeeping genes (GAPDH, ACTB, B2M, and HPRT1). Data are mean ± SEM (*n* = 6). *p < 0.05, **p < 0.01, ***p < 0.001

To determine if the GSK3-MUC signaling pathway is also impacted by modulation of B2AR and/or MOR receptor activity in vivo, we studied the effects of PRO (10 mg/kg body weight; [29]), NTX (10 mg/kg body weight; [29]), and PRO + NTX on the growth of the tumor and on the expression of genes related to GSK3-MUC1 signaling in MDA-MB-231 cell rat xenografts. Like in vitro, in rat xenografts, the growth of tumors, as determined by the tumor volume, was reduced by both PRO and NTX. The tumor growth suppression effect of these two drugs was enhanced when they were combined (Fig. 4A–E). Also, like in vitro, we found increased levels of GSK3, MUC1, CTNNB1, and caspase 3 gene expression and decreased levels of GSK3, NOTCH1, STAT3, and NKB1 expression in MDA-MB-231 cell rat xenografts following B2AR inhibition by PRO and MOR inhibition by NTX. An additive effect of these drugs on GSK3 signaling molecules was also observed in MDA-MB-231 cell rat xenografts (Fig. 4F–M).

### GSK3 may mediate B2AR and MOR regulated cell proliferation, cell migration, and colony formation in breast cancer cells

**Fig. 5:**
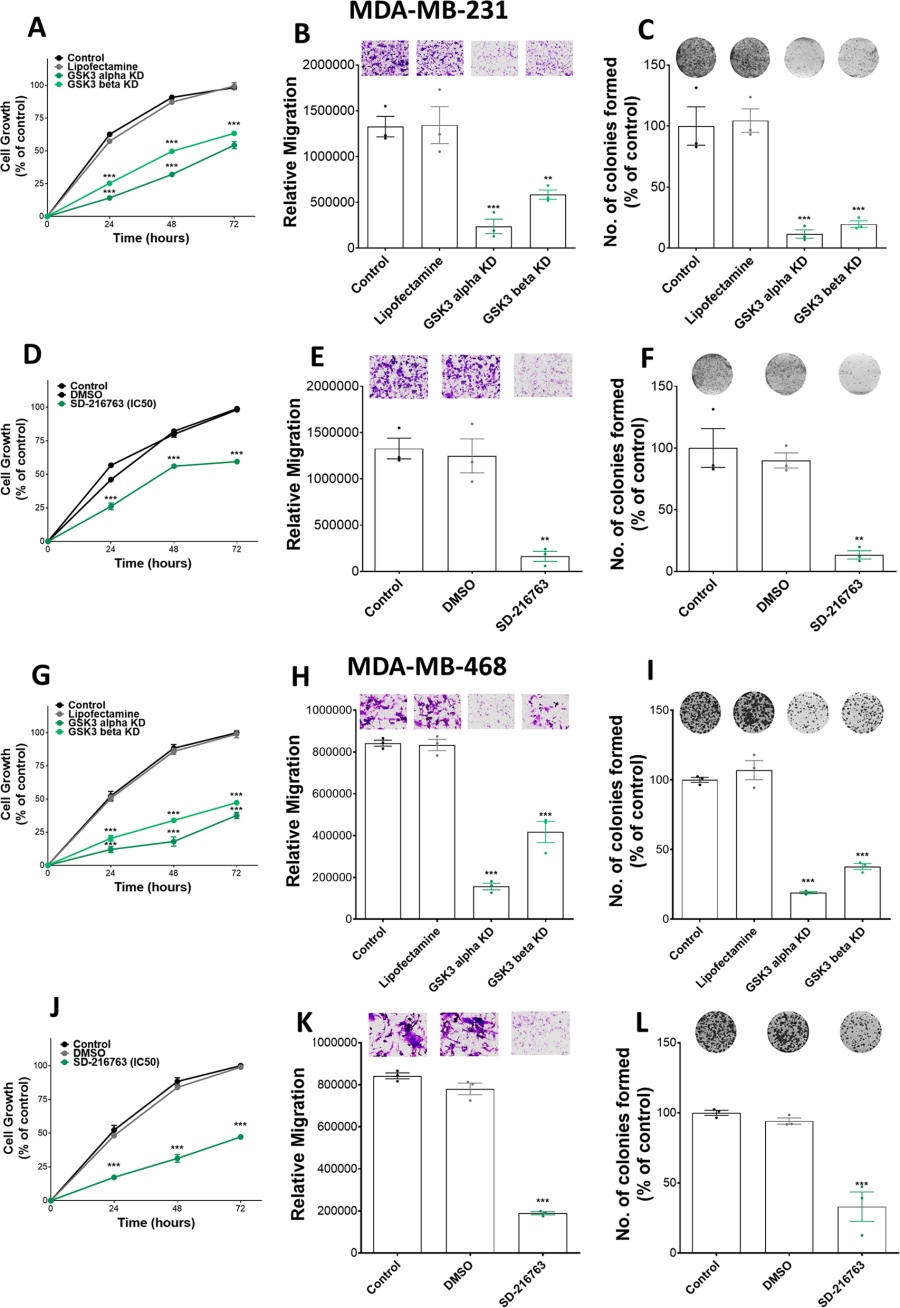
GSK3 inhibition reduces cell proliferation, cell migration, and colony formation in MDA-MB-231 and MDA-MB-468 cells. GSK3 gene expression suppression was done by CRISPR technology or pharmacological blockade by SB-216763 in MDA-MB-231 and MDA-MB-468 cells. In the pharmacological blockade study, IC50 dose (30 µM) of SB216763 was used for the cell proliferation, cell migration and colony formation studies. **A**, **D**, **G**, **J** Cell proliferation was evaluated at 0, 24, 48, and 72 h using MTT assay. **B**, **E**, **H**, **K** Migration capacity of cells was evaluated using the Transwell cell migration assay. Cells were stained with crystal violet after 3 h. Images were analyzed using ImageJ. **C**, **F**, **I**, **L** Colony formation of cells was evaluated for 2 weeks. After 14 days, colonies were stained using crystal violet. Images were analyzed using ImageJ. Data are mean ± SEM values obtained from three independent experiments. **p < 0.01, ***p < 0.001

Because suppression of GSK3 signaling is identified as a putative mechanism involved in mediation of B2AR and MOR effects on breast cancer cell growth and progression, we first tested if GSK3 gene knockdown reduces MDA-MD-231 and MDA-MB-468 cell proliferation, migration, and colony formation in vitro. We employed both gene knockdown by CRISPR and pharmacological knockdown by a specific chemical inhibitor SD-216763. In the case of gene knockdown by CRISPR, Western blot data confirmed the effectiveness of the gene knockdown (Additional file 1: Fig. S3). GSK3 gene knockdown decreased the cell proliferation level for a period of 72 h as compared to control groups (Fig. 5A, G). The migration capacity of MDA-MD-231 and MDA-MB-468 cells was markedly decreased in GSK3 knockdown cells (Fig. 5B, H, Additional file 1: Fig. S2A and S2B). Additionally, the cells with GSK3 knockdown showed a lower number of colonies compared to the controls (Fig. 5C, I, Additional file 1: Fig. S2C and S2D). In the case of pharmaceutical knockdown by SD-216763, we also found this drug is effective in inhibition of cell proliferation (Fig. 5D, J) for a period of 72 h and determined that a dose of 29.2 µM produced the half-maximal effect or IC50. The IC50 dose of SD-216763 also decreased cell migration (Fig. 5E, K, Additional file 1: Fig. S2A and S2B) and reduced the number of colony formation (Fig. 5F, L, Additional file 1: Fig. S2C and S2D) in both MDA-MB-231 and MDA-MB-468 breast cancer cell lines.

**Fig. 6:**
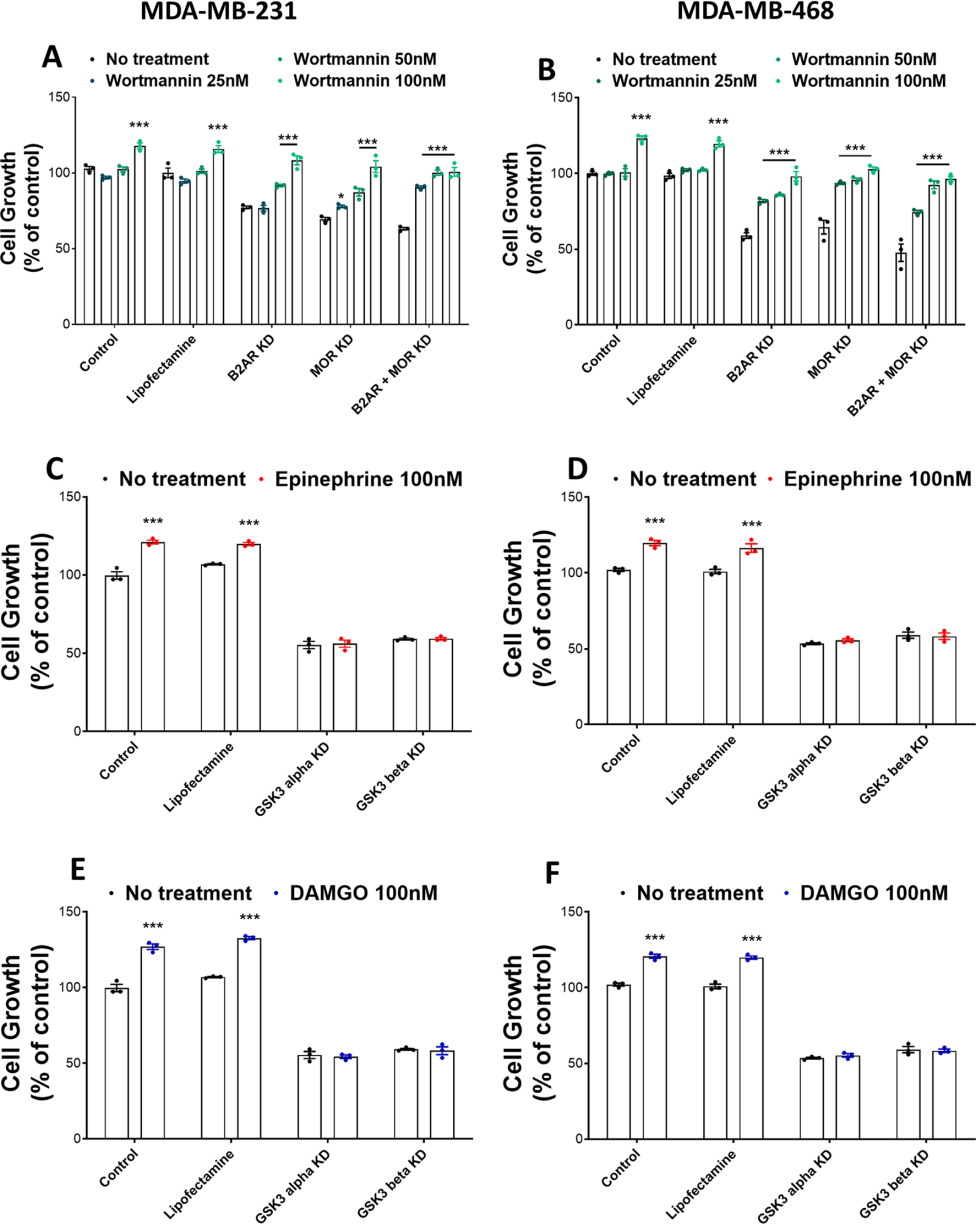
GSK3 is a downstream effector of the B2AR and MOR interactive signaling in MDA-MB-231 cells. **A**, **B** Effects of various doses of GSK3 activator wortmannin on cell proliferation responses of B2AR, MOR, and B2AR + MOR gene knockdown by CRISPR. **C**, **D** Effects of B2AR agonist epinephrine (100 nM) on cell proliferation responses of GSK3 alpha or GSK3 beta gene knockdown by CRISPR. **E**, **F** Effects of MOR agonist DAMGO (100 nM) on cell proliferation responses of GSK3 alpha or GSK3 beta gene knockdown. Lipofectamine is used as the control for CRISPR knockdown. Treatment time was 72 h. Data are mean ± SEM values obtained from three independent experiments. ***p < 0.001

To determine if GSK3 is involved in B2AR and MOR receptor signaling, we tested the effect of activation of one partner in reversing the effects of downregulation of the other partner on cell proliferation of MDA-MB-231 and MDA-MB-468 cells. Cell proliferation of untreated control, vehicle control (lipofectamine), B2AR knockdown, and MOR knockdown cells in the presence and absence of GSK3 activator wortmannin are compared and the data are presented in Fig. 6A, B. These data revealed that treatment with wortmannin dose-dependently increased % cell proliferation in control-treated cells and reversed the inhibitory effects of B2AR, MOR, and B2AR + MOR knockdown in MDA-MB-231 and MDA-MB-468 cells. We also tested the effects of a B2AR agonist epinephrine or a MOR agonist DAMGO on cell proliferation in GSK3 knockdown cells. Interestingly, B2AR agonist epinephrine at a dose of 100 nM increased the % cell proliferation in control cells but failed to alter the inhibitory effect of GSK3 knockdown in MDA-MB-231 cells (Fig. 6C) and MDA-MB-468 (Fig. 6D). Similarly, DAMGO at a dose of 100 nM increased the % cell proliferation in control cells and failed to reverse the inhibitory effect of GSK3 knockdown in MDA-MB-231 and MDA-MB-468 cells (Fig. 6E, F).

## Discussion

B2AR and MOR are G-protein-coupled receptors which are known to heterodimerize with closely related members, resulting in the modulation of their functions and signal transduction [36]. Through the inhibition by knockdown of each of the receptors, we demonstrated that the loss of expression of either of these receptors reduces the growth and the capacity to migrate and to form colonies of MDA-MB-231 and MDA-MB-468 cells. The study also identifies synergistic effects following suppression of both receptors on these cell functions. Together, these data suggest the possibility of a role of a B2AR and MOR interaction in the regulation of growth and progression of MDA-MB-231 and MDA-MB-468 cells.

A role of B2AR and MOR interaction in the regulation of cancer cell functions is further highlighted by the demonstration of expression changes in subcellular molecules involved in the regulation of cell invasion, migration, apoptosis, and cell proliferation following suppression of B2AR and/or MOR activity both by genetic modifications using CRSPR and by pharmacological approaches. Our PCR array data also identified some critical signaling pathways. Of these pathways, some noticeable pathways are NANOG signaling, a key regulator of embryonic development and cellular reprogramming that possesses protumorigenic attributes and is broadly expressed in human cancers [37]; Notch signaling, commonly activated in cancer and plays a key role in the progress of cancer [38] and has been shown to be regulated by the norepinephrine-activated β2-AR–PKA–mTOR pathway [39]; Stat3 signaling, also constitutively activated in many cancers, which regulates cellular proliferation, invasion, migration, and angiogenesis that are critical for cancer metastasis [40, 41]; and also PTEN, Wnt/b-catenin, IL-6, p53, and PI3K/AKT signaling, known to be modified in many cancers. Some of the gene expression data are also in agreement with previous findings that MOR regulates cancer cell proliferation, migration, and EMT formation by using PI3K, Akt, and STAT3 signaling [23, 42], while B2AR actions on cancer cells involve P38MAPK signaling [11, 43]. Bioinformatic analysis of the gene expression data also identifies MUC1 as the signaling molecule affected by the inhibition of both B2AR and MOR. MUC1 has been shown to be overexpressed in a variety of epithelial cancers, including breast cancer [44, 45]. Several studies have indicated that MUC1 plays a critical role in the transcriptional regulation of genes associated with tumor invasion, metastasis, angiogenesis, proliferation, apoptosis, drug resistance, inflammation, and immune regulation [31, 41, 46]. In epithelial cells, disruption of the interaction between E-cadherin and β-catenin by overexpression of MUC1 may be important in the progression to carcinoma [47]. MUC1 is shown to contribute to the malignant phenotype by preventing GSK3β-mediated phosphorylation of β-catenin and thereby stabilizes β-catenin [32]. Hence, the interaction between MUC1 and β-catenin is tightly regulated by GSK3β, and MUC1 may transiently stabilize β-catenin before GSK3β induces dissociation of the MUC1-β-catenin complex [32]. GSK3β decreases binding of β-catenin to MUC1 and stimulates the association of β-catenin and E-cadherin. We confirmed the involvement of GSK3 signaling by qRT-PCR determination of genes in B2AR and MOR knockdown cells by showing modification of the expression of different genes implicated in the pathways. Our data show a downregulation of the expression of GSK3 but an upregulation of MUC1and CTNNB1 (catenin- β], which is consistent with the view that GSK3 negatively influences the expression of MUC1 and β-catenin. In addition, we found downregulation of NOTCH1, STAT3, NF-κB, and upregulation of caspase 3. These data are consistent with several previously observed findings. For example, it has been found that a reduction in GSK-3 phosphorylation leads to a lower expression of Notch pathway members and reduces cancer cell growth [47]. Also, activation of STAT3 has been shown to be dependent upon GSK in various cell systems [48]. In addition, GSK3 has been shown to interact with NF-κB to control cellular apoptosis [49, 50]. The involvement of GSK3 signaling in the mediation of B2AR and MOR-regulated MDA-MB-231 and MDA-MB-468 cell growth and progression is further established from our data showing that the inhibition of GSK3 with chemical antagonist SD-216763 or using CRISPR technology decreased cell proliferation, cell migration, and colony formation in MDA-MB-231 and MDA-MB-468 cells. This shows that the inhibition of GSK3 in MDA-MB-231 and MDA-MB-468 cells induced the similar phenotypic changes as the inhibition of B2AR and/or MOR. In B2AR and/or MOR knockdown MDA-MB-231 and MDA-MB-468 cells, a treatment by wortmannin, agonist of GSK3, restored the proliferation rate observed in the control cells. In addition, our data indicated that activation of B2AR by norepinephrine or MOR by DAMGO has no effect on cell proliferation of GSK3 knockdown MDA-MB-231 and MDA-MB-468 cells. These data suggest that GSK3 signaling is involved in the mediation of B2AR and MOR regulated MDA-MB-231 and MDA-MB-468 cell growth and progression, possibly following dimerization of these two receptors.

Previously it has been shown that GSK3β signaling plays an important role in opioid-mediated apoptosis in breast cancer cells [51]. MOR activation by morphine has also been shown to increase phosphorylation of GSK-3β and ERK in the heart [52]. In the L6 skeletal muscle cells, it has been shown that adrenergic stimulation through β2 adrenoceptors, but not involving cyclic AMP or Gi, activates a PI3K pathway that stimulates GSK3 [53, 54]. Evidence from cultured cell studies also support interaction of B2AR with GSK3-Wnt signaling via G proteins [55]. The data presented here add the role of GSK3 in mediation of B2AR and MOR regulated TNBC cell growth and progression.

Whether or not B2AR and MOR interact similarly to affects non-TNBC cell growth and migration is not addressed in this study. In vivo studies of B2AR signaling in animal models of breast tumors demonstrated an association with increased nodal involvement and the development of tumor metastasis [56]. Also, B2AR antagonist PRO decreased proliferation, migration and invasion of TNBC and non-TNBC cells both in vitro and in vivo systems [11, 29, 57, 58]. In the case of MOR signaling, significant predominance of Ki-67 and μ-opioid receptor immuno-expression in the lymph nodes was observed in breast cancer patients with opioid medications [59]. Also, MOR antagonist naloxone or naltrexone increased the cell death while decreased the cell metastasis in an animal model of TNBC [29, 60]. In contrast, it was found that a MOR agonist morphine significantly reduced the cell vitality, growth, and colony formation rate in MCF-7 cells, but naloxone along with morphine did not reverse these effects. This indicates that the inhibition of MCF-7 cell growth and proliferation by morphine could be its independent effect, not associated with opioid receptors [61]. Further research is needed to establish if the interactive role of B2AR and MOR similarly operates to control cellular functions in both TNBC and non-TNBC tumor cells.

## Conclusions

**Fig. 7:**
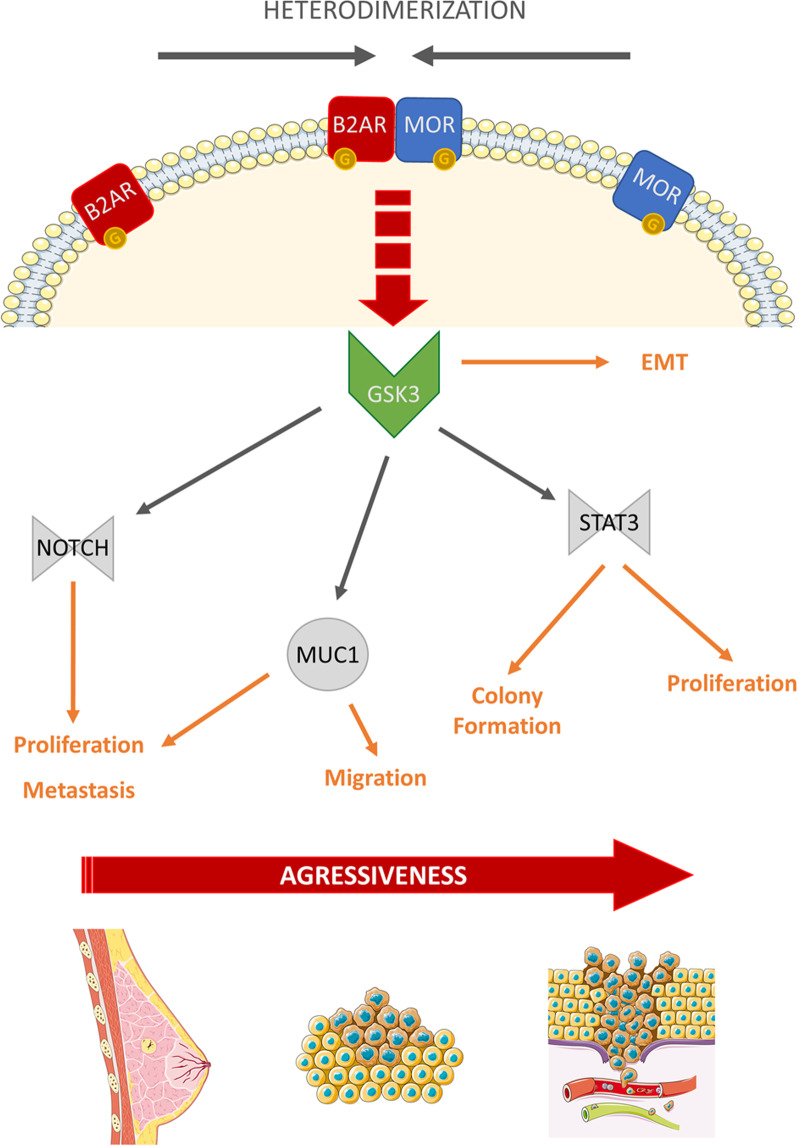
A schematic diagram showing a proposed mechanism by which beta-adrenergic receptor (B2AR) and mu-opioid receptor (MOR) interact and regulate tumor aggressiveness in human breast cancer cells. B2AR and MOR interaction via receptor dimerization increases GSK3 signaling in triple-negative MDA-MB-231 and MDA-MB-468 breast cancer cells. Increased GSK3 signaling via activation of NOTCH, MUC1, and STAT3 increases cell proliferation, colony formation, cell migration, and metastasis

Overall, we demonstrated in this study a mutual activation of B2AR and MOR through heterodimerization. The joint activation of these G protein-coupled receptors induces the activation of the tyrosine-kinase effector GSK3 to induce cell proliferation, cell migration, and colony formation possibly via alteration of MUC1, NOTCH, and STAT3 expression resulting in a more aggressive tumor profile (Fig. 7). A better understanding of the molecular mechanism implicated in the aggressiveness of a tumor led by adrenergic and opioidergic activation present B2AR, MOR, and GSK3 as potential targets for the treatment of cancers such as TNBC that are resistant to current chemotherapies.

## Supplementary Information

**Additional file 1**: Tables S1, S2, S3 and Figures S1, S2A-D, S3.

## Data Availability

All data generated or analyzed during this study are included in this published article [and its supplementary information files].

## Abbreviations

ANOVA: Analysis of variance
B2AR: Beta-adrenergic receptors
CSC: Cancer stem cells
DAMGO: [D-Ala2, N-MePhe4, Gly-ol]-enkephalin
DMEM: Dulbecco's Modified Eagle Medium
g-RNA: Guide RNA
GSK3: Glycogen synthase kinase 3
GPCRs: G-protein-coupled receptors
IPA: Ingenuity Pathway Analysis
MOR: Mu-opioid receptors
NTX: Naltrexone
PBS: Phosphate-buffered saline
PCR: Polymerase chain reaction
PRO: Propranolol
TBST: 5% Non-fat dry milk-TBS-0.1% Tween 20
TNBC: Triple-negative breast cancer
