# Supplementary material for: Beta 2 adrenergic receptor and mu opioid receptor interact to potentiate the aggressiveness of human breast cancer cell by activating the glycogen synthase kinase 3 signaling
Source: Breast Cancer Res. 2022 May 14;24:33. doi: 10.1186/s13058-022-01526-y (PMC9107672; doi:10.1186/s13058-022-01526-y)
Supplement: Supplementary file 1 — Additional file 1: Tables S1, S2, S3 and Figures S1, S2A-D, S3. [file 13058_2022_1526_MOESM1_ESM.docx]

# Supplementary Table S1

CRISPR-Cas9 Knock Down - Primers

| Targeted gene | gRNA # | CRISPR sequence | Targeted exon | Primers | |
| --- | --- | --- | --- | --- | --- |
| ADRB2 | gRNA1 | CGTCTGCAGACGCTCGAACT | 1 | F | TAATACGACTCACTATAGCGTCTGCAGACGCTCG |
|  |  |  |  | R | TTCTAGCTCTAAAACAGTTCGAGCGTCTGCAGAC |
|  | gRNA2 | CAGACGCTCGAACTTGGCAA | 1 | F | TAATACGACTCACTATAGCAGACGCTCGAACTTG |
|  |  |  |  | R | TTCTAGCTCTAAAACTTGCCAAGTTCGAGCGTCT |
|  | gRNA3 | AAGAATATGGGCGGCCCCAA | 1 | F | TAATACGACTCACTATAGAAGAATATGGGCGGCC |
|  |  |  |  | R | TTCTAGCTCTAAAACTTGGGGCCGCCCATATTCT |
| MOR | gRNA1 | CCACGCACACGATGGAGTAG | 1 | F | TAATACGACTCACTATAGCCACGCACACGATGGA |
|  |  |  |  | R | TTCTAGCTCTAAAACCTACTCCATCGTGTGCGTG |
|  | gRNA2 | CCTCTACTCCATCGTGTGCG | 1 | F | TAATACGACTCACTATAGCCTCTACTCCATCGTG |
|  |  |  |  | R | TTCTAGCTCTAAAACCGCACACGATGGAGTAGAG |
|  | gRNA3 | TCGGGGAGTACGGAAATCTA | 2 | F | TAATACGACTCACTATAGTCGGGGAGTACGGAAA |
|  |  |  |  | R | TTCTAGCTCTAAAACTAGATTTCCGTACTCCCCG |
| GSK3 alpha | gRNA1 | GTCCGTGTAAGCCACTTCTT | 1 | F | TAATACGACTCACTATAGGATCTAGCTTTCTCAT |
|  |  |  |  | R | TTCTAGCTCTAAAACGATCATGAGAAAGCTAGAT |
|  | gRNA2 | TGTCCGTGTAAGCCACTTCT | 1 | F | TAATACGACTCACTATAGCGCCATCAAGAAAGTA |
|  |  |  |  | R | TTCTAGCTCTAAAACGCAATACTTTCTTGATGGC |
|  | gRNA3 | GCCTAGAGTGGCTACGACTG | 1 | F | TAATACGACTCACTATAGTCCGCAAAGGAGGTGG |
|  |  |  |  | R | TTCTAGCTCTAAAACAGAACCACCTCCTTTGCGG |
| GSK3 beta | gRNA1 | GATCTAGCTTTCTCATGATC | 3 | F | TAATACGACTCACTATAGGATCTAGCTTTCTCAT |
|  |  |  |  | R | TTCTAGCTCTAAAACGATCATGAGAAAGCTAGAT |
|  | gRNA2 | CGCCATCAAGAAAGTATTGC | 2 | F | TAATACGACTCACTATAGCGCCATCAAGAAAGTA |
|  |  |  |  | R | TTCTAGCTCTAAAACGCAATACTTTCTTGATGGC |
|  | gRNA3 | GTCCTGCAATACTTTCTTGA | 2 | F | TAATACGACTCACTATAGTCCGCAAAGGAGGTGG |
|  |  |  |  | R | TTCTAGCTCTAAAACAGAACCACCTCCTTTGCGG |

# Supplementary Table S2

Cancer Stem Cells genes qPCR Array – genes list

|  | 1 | 2 | 3 | 4 | 5 | 6 | 7 | 8 | 9 | 10 | 11 | 12 |
| --- | --- | --- | --- | --- | --- | --- | --- | --- | --- | --- | --- | --- |
| A | ABCB5 | ABCG2 | ALCAM | ALDH1A1 | ATM | ATXN1 | AXL | BMI1 | BMP7 | CD24 | CD34 | CD38 |
| B | CD44 | CHEK1 | DACH1 | DDR1 | DKK1 | DLL1 | DLL4 | DNMT1 | EGF | ENG | EPCAM | ERBB2 |
| C | ETFA | FGFR2 | FLOT2 | FOXA2 | FOXP1 | FZD7 | GATA3 | GSK3B | HDAC1 | ID1 | IKBKB | IL8 |
| D | ITGA2 | ITGA4 | ITGA6 | ITGB1 | JAG1 | JAK2 | KIT | KITLG | KLF17 | KLF4 | LATS1 | LIN28A |
| E | LIN28B | MAML1 | MERTK | MS4A1 | MUC1 | MYC | MYCN | NANOG | NFKB1 | NOS2 | NOTCH1 | NOTCH2 |
| F | PECAM1 | PLAT | PLAUR | POU5F1 | PROM1 | PTCH1 | PTPRC | SAV1 | SIRT1 | SMO | SNAI1 | SOX2 |
| G | STAT3 | TAZ | TGFBR1 | THY1 | TWIST1 | TWIST2 | WEE1 | WNT1 | WWC1 | YAP1 | ZEB1 | ZEB2 |
| H | ACTB | B2M | GAPDH | HPRT1 | RPLP0 | HGDC | RTC | RTC | RTC | PPC | PPC | PPC |

**Cancer Stem Cell Markers**
[ABCB5](http://www.ncbi.nlm.nih.gov/entrez/query.fcgi?db=gene&cmd=Retrieve&dopt=Graphics&list_uids=340273), [ALCAM](http://www.ncbi.nlm.nih.gov/entrez/query.fcgi?db=gene&cmd=Retrieve&dopt=Graphics&list_uids=214), [ALDH1A1](http://www.ncbi.nlm.nih.gov/entrez/query.fcgi?db=gene&cmd=Retrieve&dopt=Graphics&list_uids=216) (RALDH1), [ATXN1](http://www.ncbi.nlm.nih.gov/entrez/query.fcgi?db=gene&cmd=Retrieve&dopt=Graphics&list_uids=6310), [BMI1](http://www.ncbi.nlm.nih.gov/entrez/query.fcgi?db=gene&cmd=Retrieve&dopt=Graphics&list_uids=648) (PCGF4), [CD24](http://www.ncbi.nlm.nih.gov/entrez/query.fcgi?db=gene&cmd=Retrieve&dopt=Graphics&list_uids=100133941), [CD34](http://www.ncbi.nlm.nih.gov/entrez/query.fcgi?db=gene&cmd=Retrieve&dopt=Graphics&list_uids=947), [CD38](http://www.ncbi.nlm.nih.gov/entrez/query.fcgi?db=gene&cmd=Retrieve&dopt=Graphics&list_uids=952), [CD44](http://www.ncbi.nlm.nih.gov/entrez/query.fcgi?db=gene&cmd=Retrieve&dopt=Graphics&list_uids=960), [ENG](http://www.ncbi.nlm.nih.gov/entrez/query.fcgi?db=gene&cmd=Retrieve&dopt=Graphics&list_uids=2022) (EVI-1), [ETFA](http://www.ncbi.nlm.nih.gov/entrez/query.fcgi?db=gene&cmd=Retrieve&dopt=Graphics&list_uids=2108), [FLOT2](http://www.ncbi.nlm.nih.gov/entrez/query.fcgi?db=gene&cmd=Retrieve&dopt=Graphics&list_uids=2319), [GATA3](http://www.ncbi.nlm.nih.gov/entrez/query.fcgi?db=gene&cmd=Retrieve&dopt=Graphics&list_uids=2625), [ITGA2](http://www.ncbi.nlm.nih.gov/entrez/query.fcgi?db=gene&cmd=Retrieve&dopt=Graphics&list_uids=3673), [ITGA4](http://www.ncbi.nlm.nih.gov/entrez/query.fcgi?db=gene&cmd=Retrieve&dopt=Graphics&list_uids=3676) (CD49D), [ITGA6](http://www.ncbi.nlm.nih.gov/entrez/query.fcgi?db=gene&cmd=Retrieve&dopt=Graphics&list_uids=3655), [ITGB1](http://www.ncbi.nlm.nih.gov/entrez/query.fcgi?db=gene&cmd=Retrieve&dopt=Graphics&list_uids=3688), [KIT](http://www.ncbi.nlm.nih.gov/entrez/query.fcgi?db=gene&cmd=Retrieve&dopt=Graphics&list_uids=3815) (CD117), [MS4A1](http://www.ncbi.nlm.nih.gov/entrez/query.fcgi?db=gene&cmd=Retrieve&dopt=Graphics&list_uids=931), [MUC1](http://www.ncbi.nlm.nih.gov/entrez/query.fcgi?db=gene&cmd=Retrieve&dopt=Graphics&list_uids=4582) (mucin), [PECAM1](http://www.ncbi.nlm.nih.gov/entrez/query.fcgi?db=gene&cmd=Retrieve&dopt=Graphics&list_uids=5175), [PROM1](http://www.ncbi.nlm.nih.gov/entrez/query.fcgi?db=gene&cmd=Retrieve&dopt=Graphics&list_uids=8842), [PTPRC](http://www.ncbi.nlm.nih.gov/entrez/query.fcgi?db=gene&cmd=Retrieve&dopt=Graphics&list_uids=5788), [THY1](http://www.ncbi.nlm.nih.gov/entrez/query.fcgi?db=gene&cmd=Retrieve&dopt=Graphics&list_uids=7070).

**Cell Proliferation**
[EGF](http://www.ncbi.nlm.nih.gov/entrez/query.fcgi?db=gene&cmd=Retrieve&dopt=Graphics&list_uids=1950), [ERBB2](http://www.ncbi.nlm.nih.gov/entrez/query.fcgi?db=gene&cmd=Retrieve&dopt=Graphics&list_uids=2064) (HER-2, NEU), [KITLG](http://www.ncbi.nlm.nih.gov/entrez/query.fcgi?db=gene&cmd=Retrieve&dopt=Graphics&list_uids=4254) (SCF), [LIN28B](http://www.ncbi.nlm.nih.gov/entrez/query.fcgi?db=gene&cmd=Retrieve&dopt=Graphics&list_uids=389421), [NOS2](http://www.ncbi.nlm.nih.gov/entrez/query.fcgi?db=gene&cmd=Retrieve&dopt=Graphics&list_uids=4843) (INOS).

**Self-Renewal**
[BMP7](http://www.ncbi.nlm.nih.gov/entrez/query.fcgi?db=gene&cmd=Retrieve&dopt=Graphics&list_uids=655), [DNMT1](http://www.ncbi.nlm.nih.gov/entrez/query.fcgi?db=gene&cmd=Retrieve&dopt=Graphics&list_uids=1786), [FGFR2](http://www.ncbi.nlm.nih.gov/entrez/query.fcgi?db=gene&cmd=Retrieve&dopt=Graphics&list_uids=2263).

**Pluripotency**
[KLF4](http://www.ncbi.nlm.nih.gov/entrez/query.fcgi?db=gene&cmd=Retrieve&dopt=Graphics&list_uids=9314), [LIN28A](http://www.ncbi.nlm.nih.gov/entrez/query.fcgi?db=gene&cmd=Retrieve&dopt=Graphics&list_uids=79727), [MYC](http://www.ncbi.nlm.nih.gov/entrez/query.fcgi?db=gene&cmd=Retrieve&dopt=Graphics&list_uids=4609), [NANOG](http://www.ncbi.nlm.nih.gov/entrez/query.fcgi?db=gene&cmd=Retrieve&dopt=Graphics&list_uids=79923), [POU5F1](http://www.ncbi.nlm.nih.gov/entrez/query.fcgi?db=gene&cmd=Retrieve&dopt=Graphics&list_uids=5460) (Oct4), [SOX2](http://www.ncbi.nlm.nih.gov/entrez/query.fcgi?db=gene&cmd=Retrieve&dopt=Graphics&list_uids=6657).

**Asymmetric Division**
[FOXP1](http://www.ncbi.nlm.nih.gov/entrez/query.fcgi?db=gene&cmd=Retrieve&dopt=Graphics&list_uids=27086), [HDAC1](http://www.ncbi.nlm.nih.gov/entrez/query.fcgi?db=gene&cmd=Retrieve&dopt=Graphics&list_uids=3065), [MYCN](http://www.ncbi.nlm.nih.gov/entrez/query.fcgi?db=gene&cmd=Retrieve&dopt=Graphics&list_uids=4613), [SIRT1](http://www.ncbi.nlm.nih.gov/entrez/query.fcgi?db=gene&cmd=Retrieve&dopt=Graphics&list_uids=23411), [WNT1](http://www.ncbi.nlm.nih.gov/entrez/query.fcgi?db=gene&cmd=Retrieve&dopt=Graphics&list_uids=7471).

**Cell Migration & Metastasis**
[AXL](http://www.ncbi.nlm.nih.gov/entrez/query.fcgi?db=gene&cmd=Retrieve&dopt=Graphics&list_uids=558), [ID1](http://www.ncbi.nlm.nih.gov/entrez/query.fcgi?db=gene&cmd=Retrieve&dopt=Graphics&list_uids=3397), [CXCL8](http://www.ncbi.nlm.nih.gov/entrez/query.fcgi?db=gene&cmd=Retrieve&dopt=Graphics&list_uids=3576), [KLF17](http://www.ncbi.nlm.nih.gov/entrez/query.fcgi?db=gene&cmd=Retrieve&dopt=Graphics&list_uids=128209), [PLAT](http://www.ncbi.nlm.nih.gov/entrez/query.fcgi?db=gene&cmd=Retrieve&dopt=Graphics&list_uids=5327) (TPA), [PLAUR](http://www.ncbi.nlm.nih.gov/entrez/query.fcgi?db=gene&cmd=Retrieve&dopt=Graphics&list_uids=5329) (UPAR), [SNAI1](http://www.ncbi.nlm.nih.gov/entrez/query.fcgi?db=gene&cmd=Retrieve&dopt=Graphics&list_uids=6615) (SNAIL), [TWIST1](http://www.ncbi.nlm.nih.gov/entrez/query.fcgi?db=gene&cmd=Retrieve&dopt=Graphics&list_uids=7291), [TWIST2](http://www.ncbi.nlm.nih.gov/entrez/query.fcgi?db=gene&cmd=Retrieve&dopt=Graphics&list_uids=117581), [ZEB1](http://www.ncbi.nlm.nih.gov/entrez/query.fcgi?db=gene&cmd=Retrieve&dopt=Graphics&list_uids=6935), [ZEB2](http://www.ncbi.nlm.nih.gov/entrez/query.fcgi?db=gene&cmd=Retrieve&dopt=Graphics&list_uids=9839).

**Loss of Stemness**
[ALDH1A1](http://www.ncbi.nlm.nih.gov/entrez/query.fcgi?db=gene&cmd=Retrieve&dopt=Graphics&list_uids=216) (RALDH1), [CD34](http://www.ncbi.nlm.nih.gov/entrez/query.fcgi?db=gene&cmd=Retrieve&dopt=Graphics&list_uids=947), [DACH1](http://www.ncbi.nlm.nih.gov/entrez/query.fcgi?db=gene&cmd=Retrieve&dopt=Graphics&list_uids=1602), [FOXA2](http://www.ncbi.nlm.nih.gov/entrez/query.fcgi?db=gene&cmd=Retrieve&dopt=Graphics&list_uids=3170) (HNF3B), [PECAM1](http://www.ncbi.nlm.nih.gov/entrez/query.fcgi?db=gene&cmd=Retrieve&dopt=Graphics&list_uids=5175), [PTCH1](http://www.ncbi.nlm.nih.gov/entrez/query.fcgi?db=gene&cmd=Retrieve&dopt=Graphics&list_uids=5727).

**Signal Transduction**
Hippo Signaling: [LATS1](http://www.ncbi.nlm.nih.gov/entrez/query.fcgi?db=gene&cmd=Retrieve&dopt=Graphics&list_uids=9113), [MERTK](http://www.ncbi.nlm.nih.gov/entrez/query.fcgi?db=gene&cmd=Retrieve&dopt=Graphics&list_uids=10461), [SAV1](http://www.ncbi.nlm.nih.gov/entrez/query.fcgi?db=gene&cmd=Retrieve&dopt=Graphics&list_uids=60485), [TAZ](http://www.ncbi.nlm.nih.gov/entrez/query.fcgi?db=gene&cmd=Retrieve&dopt=Graphics&list_uids=6901), [WWC1](http://www.ncbi.nlm.nih.gov/entrez/query.fcgi?db=gene&cmd=Retrieve&dopt=Graphics&list_uids=23286), [YAP1](http://www.ncbi.nlm.nih.gov/entrez/query.fcgi?db=gene&cmd=Retrieve&dopt=Graphics&list_uids=10413).
Hedgehog Signaling: [PTCH1](http://www.ncbi.nlm.nih.gov/entrez/query.fcgi?db=gene&cmd=Retrieve&dopt=Graphics&list_uids=5727), [SMO](http://www.ncbi.nlm.nih.gov/entrez/query.fcgi?db=gene&cmd=Retrieve&dopt=Graphics&list_uids=6608).
Notch Signaling: [DLL1](http://www.ncbi.nlm.nih.gov/entrez/query.fcgi?db=gene&cmd=Retrieve&dopt=Graphics&list_uids=28514) (DELTA1), [DLL4](http://www.ncbi.nlm.nih.gov/entrez/query.fcgi?db=gene&cmd=Retrieve&dopt=Graphics&list_uids=54567), [JAG1](http://www.ncbi.nlm.nih.gov/entrez/query.fcgi?db=gene&cmd=Retrieve&dopt=Graphics&list_uids=182), [MAML1](http://www.ncbi.nlm.nih.gov/entrez/query.fcgi?db=gene&cmd=Retrieve&dopt=Graphics&list_uids=9794), [NOTCH1](http://www.ncbi.nlm.nih.gov/entrez/query.fcgi?db=gene&cmd=Retrieve&dopt=Graphics&list_uids=4851), [NOTCH2](http://www.ncbi.nlm.nih.gov/entrez/query.fcgi?db=gene&cmd=Retrieve&dopt=Graphics&list_uids=4853).
WNT Signaling: [DKK1](http://www.ncbi.nlm.nih.gov/entrez/query.fcgi?db=gene&cmd=Retrieve&dopt=Graphics&list_uids=22943), [EPCAM](http://www.ncbi.nlm.nih.gov/entrez/query.fcgi?db=gene&cmd=Retrieve&dopt=Graphics&list_uids=4072), [FZD7](http://www.ncbi.nlm.nih.gov/entrez/query.fcgi?db=gene&cmd=Retrieve&dopt=Graphics&list_uids=8324), [WNT1](http://www.ncbi.nlm.nih.gov/entrez/query.fcgi?db=gene&cmd=Retrieve&dopt=Graphics&list_uids=7471).
AKT & PI3 Kinase / mTOR Signaling: [ABCG2](http://www.ncbi.nlm.nih.gov/entrez/query.fcgi?db=gene&cmd=Retrieve&dopt=Graphics&list_uids=9429) (BCRP), [GSK3B](http://www.ncbi.nlm.nih.gov/entrez/query.fcgi?db=gene&cmd=Retrieve&dopt=Graphics&list_uids=2932).
STAT / NFκB Signaling: [IKBKB](http://www.ncbi.nlm.nih.gov/entrez/query.fcgi?db=gene&cmd=Retrieve&dopt=Graphics&list_uids=3551) (IKKβ), [JAK2](http://www.ncbi.nlm.nih.gov/entrez/query.fcgi?db=gene&cmd=Retrieve&dopt=Graphics&list_uids=3717), [NFKB1](http://www.ncbi.nlm.nih.gov/entrez/query.fcgi?db=gene&cmd=Retrieve&dopt=Graphics&list_uids=4790).

**Cancer Therapeutic Targets**
[ABCG2](http://www.ncbi.nlm.nih.gov/entrez/query.fcgi?db=gene&cmd=Retrieve&dopt=Graphics&list_uids=9429) (BCRP), [ATM](http://www.ncbi.nlm.nih.gov/entrez/query.fcgi?db=gene&cmd=Retrieve&dopt=Graphics&list_uids=472), [AXL](http://www.ncbi.nlm.nih.gov/entrez/query.fcgi?db=gene&cmd=Retrieve&dopt=Graphics&list_uids=558), [CHEK1](http://www.ncbi.nlm.nih.gov/entrez/query.fcgi?db=gene&cmd=Retrieve&dopt=Graphics&list_uids=1111), [DDR1](http://www.ncbi.nlm.nih.gov/entrez/query.fcgi?db=gene&cmd=Retrieve&dopt=Graphics&list_uids=780), [DKK1](http://www.ncbi.nlm.nih.gov/entrez/query.fcgi?db=gene&cmd=Retrieve&dopt=Graphics&list_uids=22943), [EPCAM](http://www.ncbi.nlm.nih.gov/entrez/query.fcgi?db=gene&cmd=Retrieve&dopt=Graphics&list_uids=4072), [FZD7](http://www.ncbi.nlm.nih.gov/entrez/query.fcgi?db=gene&cmd=Retrieve&dopt=Graphics&list_uids=8324), [GSK3B](http://www.ncbi.nlm.nih.gov/entrez/query.fcgi?db=gene&cmd=Retrieve&dopt=Graphics&list_uids=2932), [ID1](http://www.ncbi.nlm.nih.gov/entrez/query.fcgi?db=gene&cmd=Retrieve&dopt=Graphics&list_uids=3397), [IKBKB](http://www.ncbi.nlm.nih.gov/entrez/query.fcgi?db=gene&cmd=Retrieve&dopt=Graphics&list_uids=3551) (IKKβ), [JAK2](http://www.ncbi.nlm.nih.gov/entrez/query.fcgi?db=gene&cmd=Retrieve&dopt=Graphics&list_uids=3717), [KLF17](http://www.ncbi.nlm.nih.gov/entrez/query.fcgi?db=gene&cmd=Retrieve&dopt=Graphics&list_uids=128209), [NFKB1](http://www.ncbi.nlm.nih.gov/entrez/query.fcgi?db=gene&cmd=Retrieve&dopt=Graphics&list_uids=4790), [PTCH1](http://www.ncbi.nlm.nih.gov/entrez/query.fcgi?db=gene&cmd=Retrieve&dopt=Graphics&list_uids=5727), [SMO](http://www.ncbi.nlm.nih.gov/entrez/query.fcgi?db=gene&cmd=Retrieve&dopt=Graphics&list_uids=6608), [STAT3](http://www.ncbi.nlm.nih.gov/entrez/query.fcgi?db=gene&cmd=Retrieve&dopt=Graphics&list_uids=6774), [TGFBR1](http://www.ncbi.nlm.nih.gov/entrez/query.fcgi?db=gene&cmd=Retrieve&dopt=Graphics&list_uids=7046) (ALK5), [WEE1](http://www.ncbi.nlm.nih.gov/entrez/query.fcgi?db=gene&cmd=Retrieve&dopt=Graphics&list_uids=7465).

# Supplementary Table S3

qPCR - Primers

| Gene name |  | Primers sequence |
| --- | --- | --- |
| GSK3 alpha | F | GGAGCCCAATGTCTCCTACA |
|  | R | GTACACAGCCAGCTGACCAA |
| GSK3 beta | F | AGGATTCGTCAGGAACAGGA |
|  | R | GTGTTAGTCGGGCAGTTGGT |
| NOTCH1 | F | GACCTCATCAACTCACACGC |
|  | R | TTAGCCCCGTTCTTCAGGAG |
| STAT3 | F | TTGCGTGTCTAAAGGTCCCT |
|  | R | GCCCCATAGTGTGCATCATG |
| CTNNB1 | F | TCATGCGTTCTCCTCAGATG |
|  | R | CTCACGATGATGGGAAAGGT |
| NFKB1 | F | CCAGCTGGCAGGTATTTGAC |
|  | R | TGCAGCTTCACATCTTCAGC |
| Caspase3 | F | GCTGAGCTGCCTGTAACTTG |
|  | R | GCGTATGGAGAAATGGGCTG |
| MUC1 | F | CTCATTGCCTTGGCTGTCTG |
|  | R | GTGTGGTAGGTGGGGTACTC |
| GAPDH | F | ACCCAGAAGACTGTGGATGG |
|  | R | TTCAGCTCAGGGATGACCTT |
| ACTB | F | CTCTTCCAGCCTTCCTTCCT |
|  | R | AGCACTGTGTTGGCGTACAG |
| B2M | F | AGGCTATCCAGCGTACTCCA |
|  | R | TCAATGTCGGATGGATGAAA |
| HPRT1 | F | GACCAGTCAACAGGGGACAT |
|  | R | CTGCATTGTTTTGCCAGTGT |

**Figure 1**

#
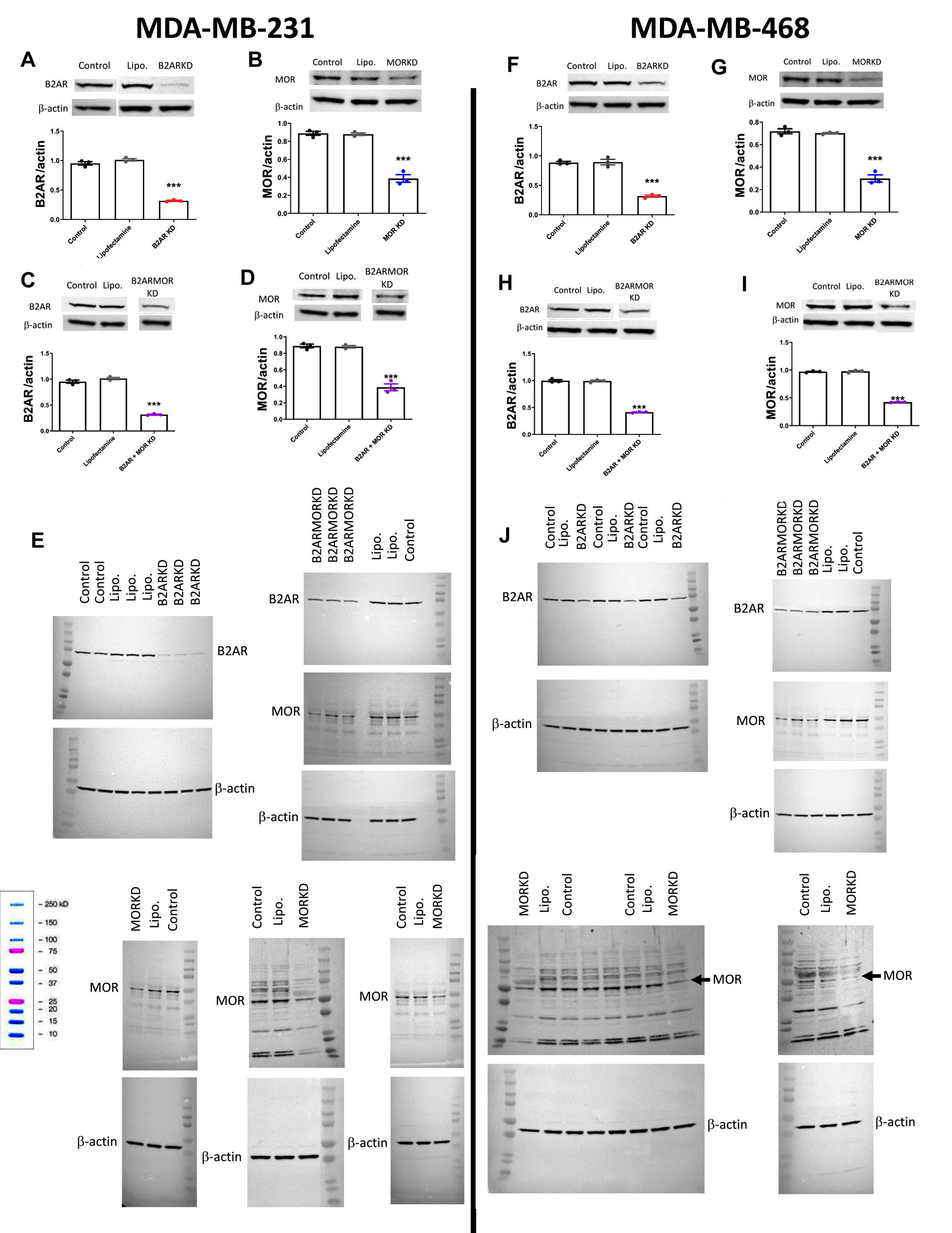


# Fig. S1. Western blot analysis represents successful B2AR and/or MOR knockdown in MDA-MB-231 and MDA-MB-468 cells. CRISPR knockdown of B2AR and/or MOR receptors in MDA-MB-231 (A,B,C,D, E) and MDA-MB-468 (F,G,H,I,J) cells were validated using western blotting. Immediately following transfection, some cells were extracted and used for Western blot analysis of B2AR and/or MOR. Representative blots are presented on the top and mean densitometric values are presented as ratio of β-actin in the histograms. Data are mean ± SEM values of three independent experiments. *** p < 0.001. Individual blots and protein markers are shown in E and J.

**Figure S2A**


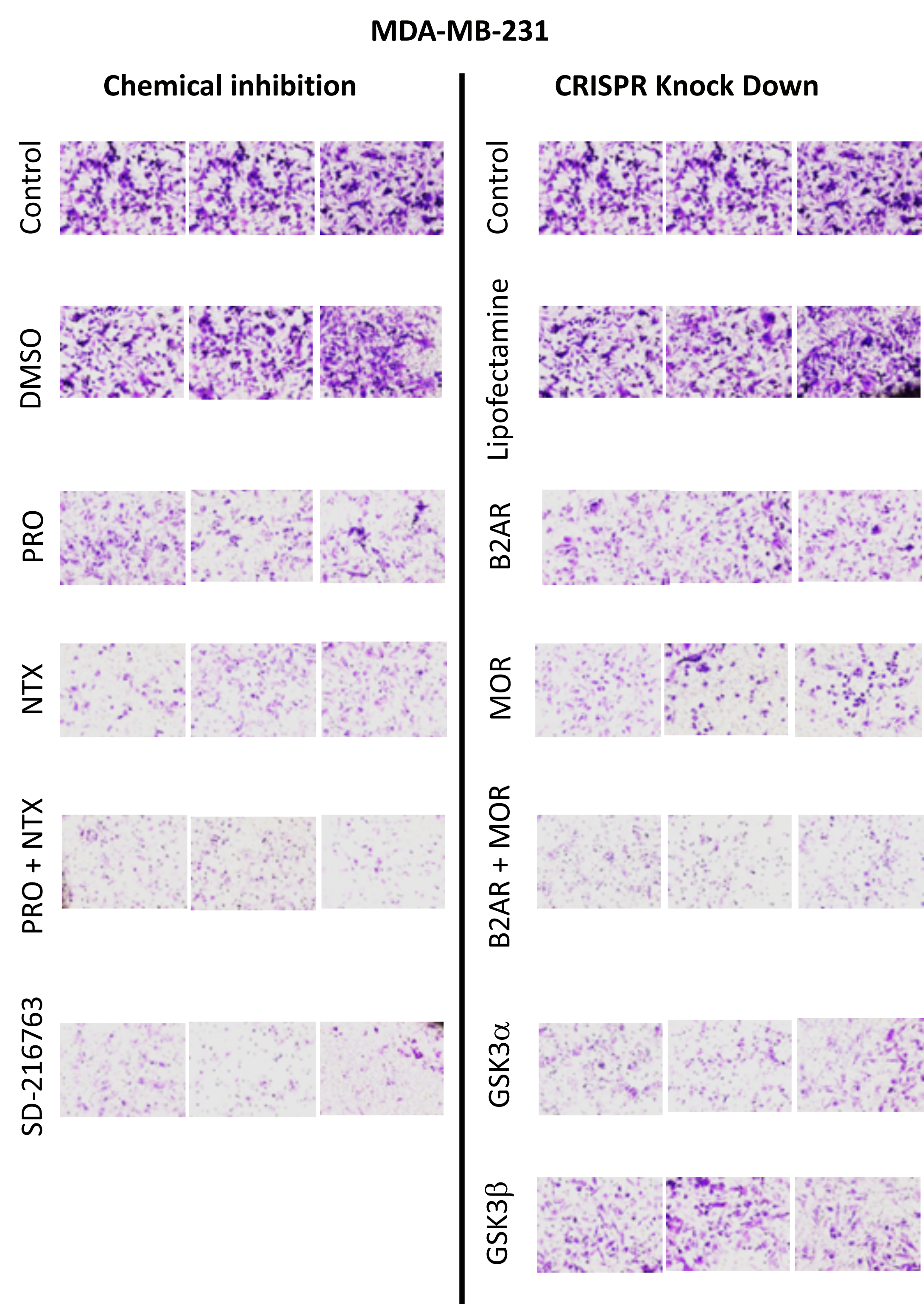


**Figure S2B**

**
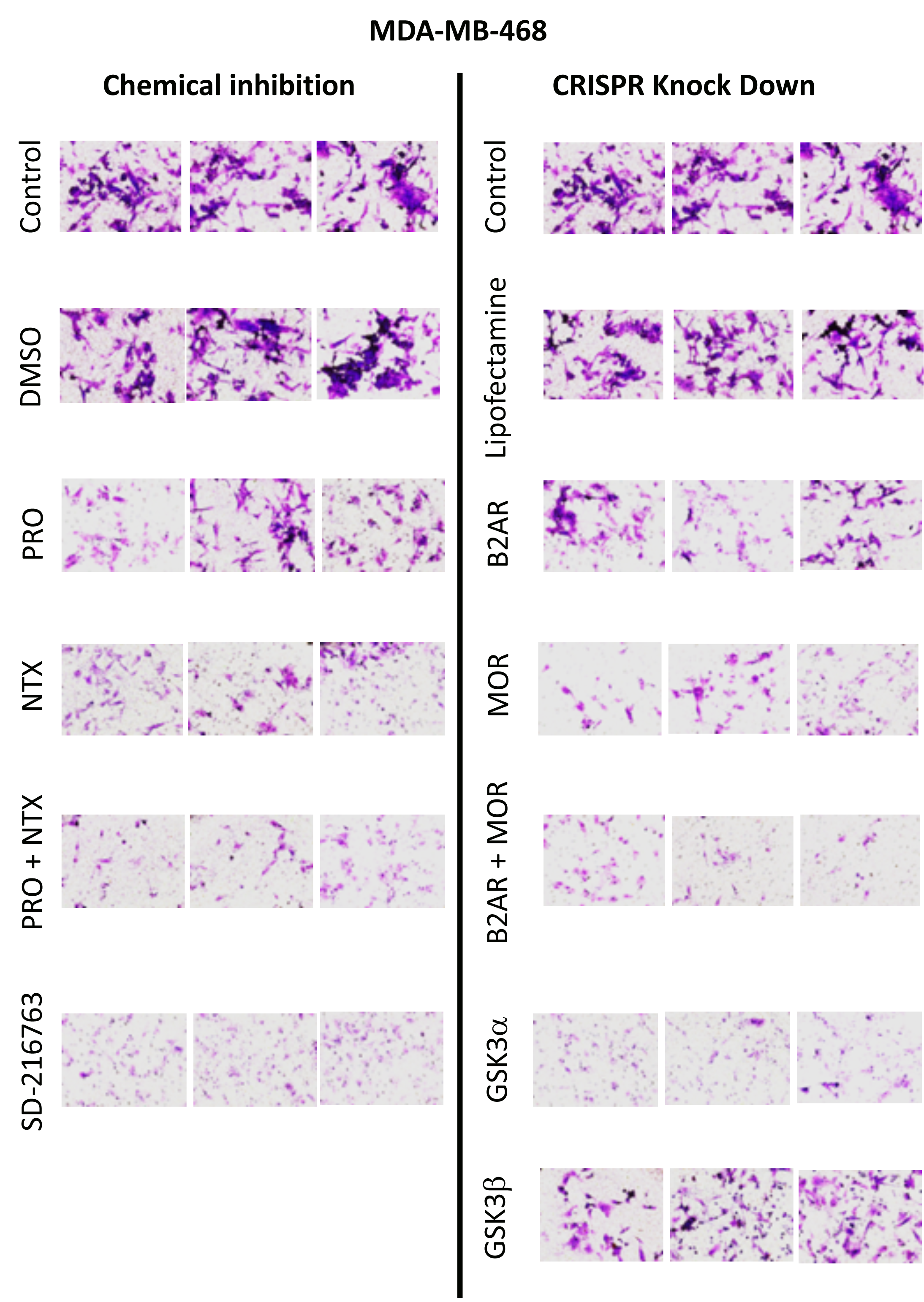
**

**Figure S2C**

**
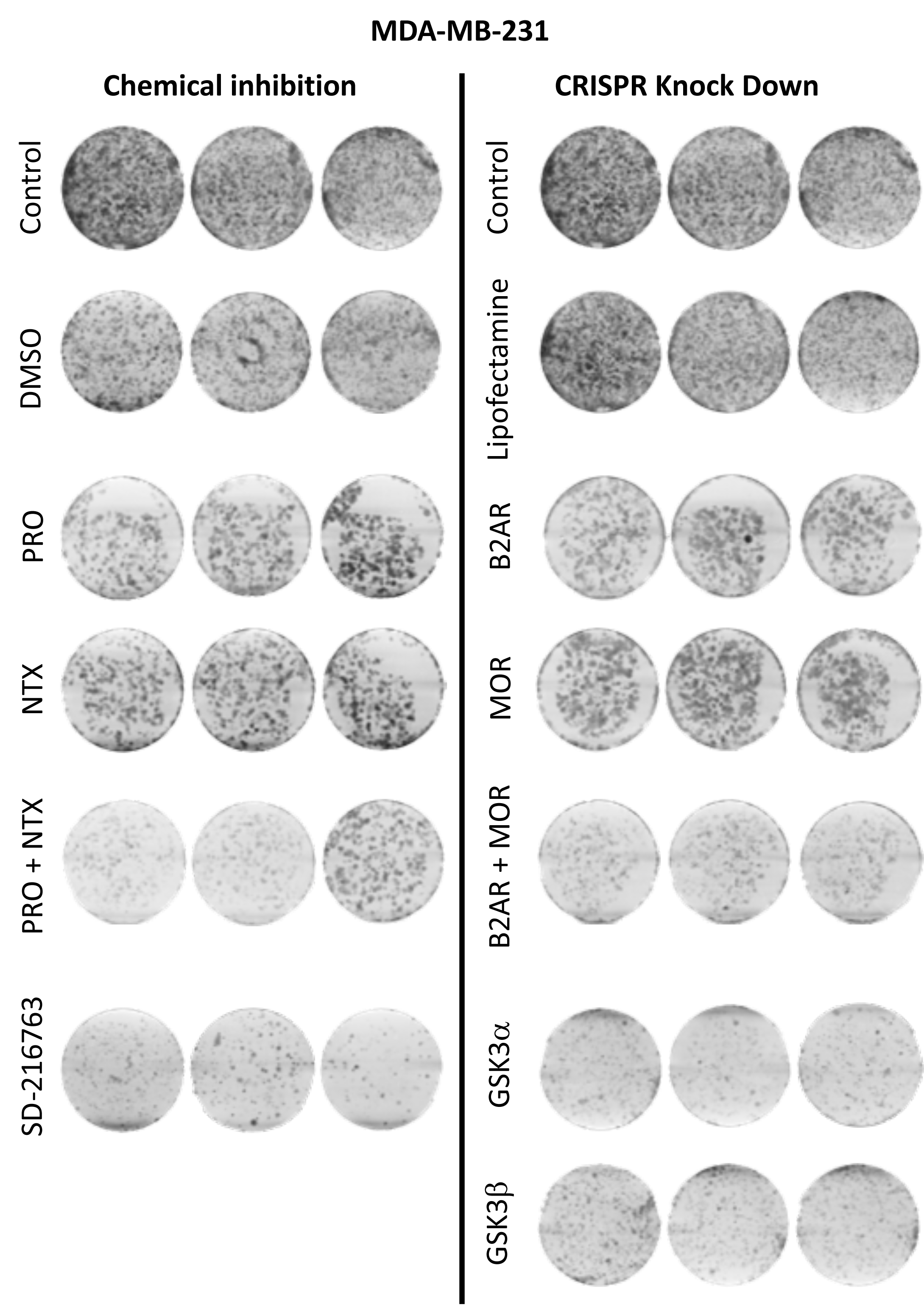
**

**Figure S2D**

**
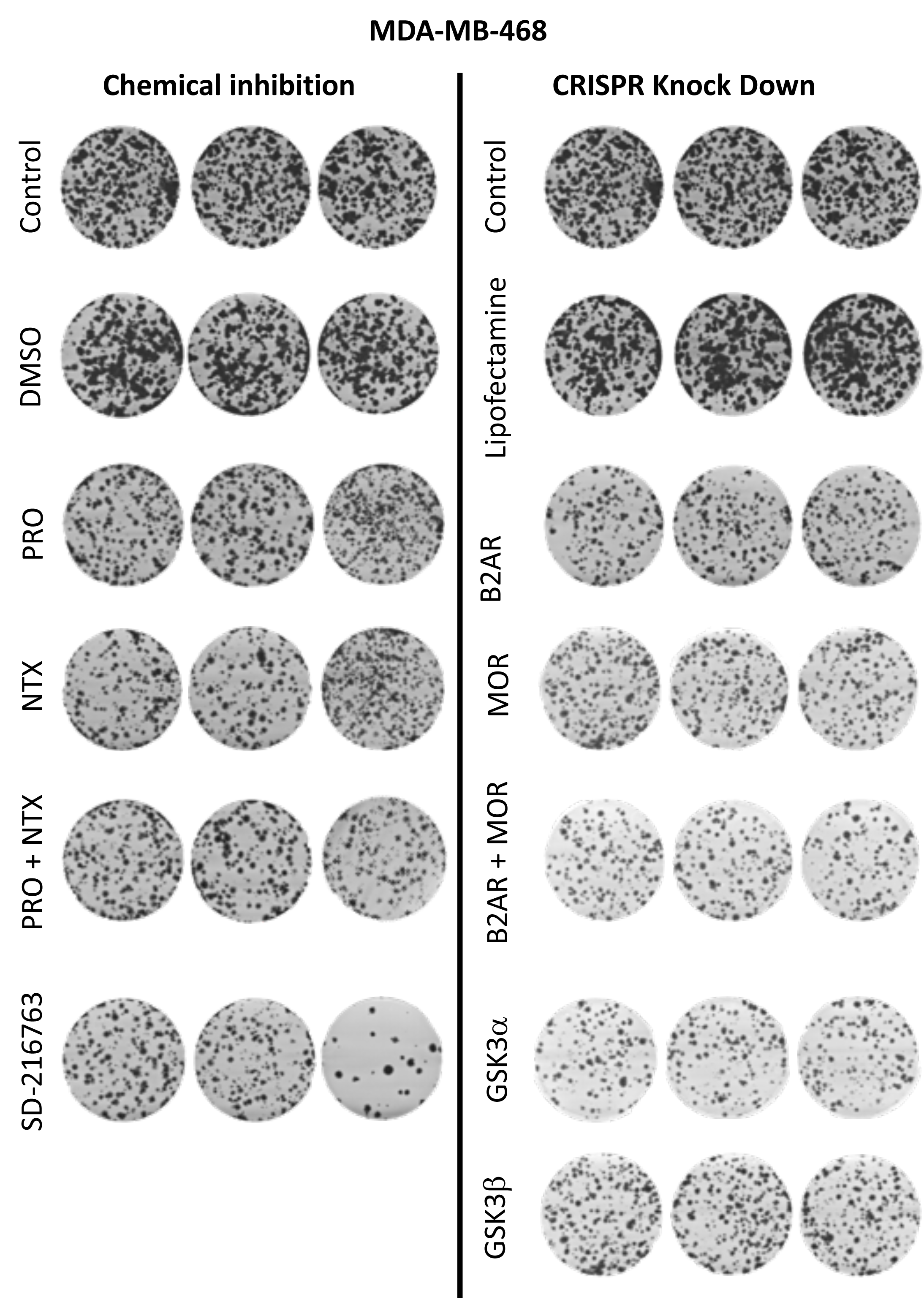
**

**Figure S2.** **B2AR, MOR and GSK3 knockdown reduces cell migration and colony formation in breast cancer cells**. B2AR and/or MOR, GSK3⍺ or GSK3β gene expression suppression was done by CRISPR technology or pharmacological blockade of PRO (100 µM), NTX (100 µM), PRO+NTX, or SD-216763 in MDA-MB-231 and MDA-MB-468 cells. Control groups received media only (Control) or Lipofectamine only (Lipofectamine). (A, B) Cell migration capacity was evaluated using the Transwell cell migration assay as described in materials and methods. Cells were stained with crystal violet after 3 hours. Representative images for MDA-MB-231 cells (A) and MDA-MB-468 (B) cells are shown. Images were analyzed using ImageJ. (C, D) Colony formation by cells was evaluated for 2 weeks as described in materials and methods. After 14 days, colonies were stained using crystal violet. Images were analyzed using ImageJ. Representative images are shown here for MDA-MB-231 cells (G) and MDA-MB-468 cells (H). The mean ± SEM values of cell migration and colony formation data are presented in figure 1B, C, E, F, H, I, K, L, and figure 5B, C, E, F, H, I, K, L.

# Figure S3


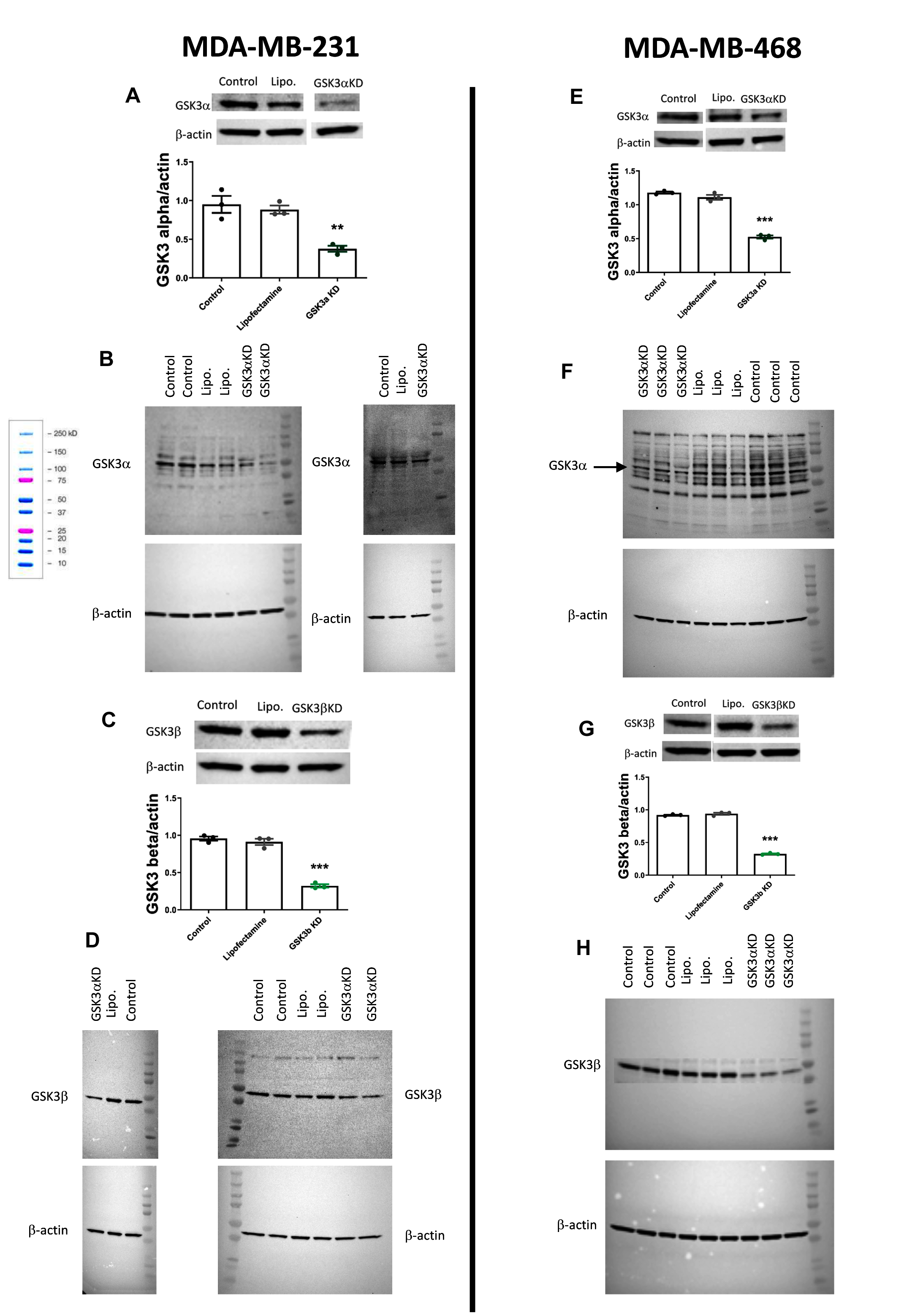


# Fig. S3. Western blot analysis represents successful GSK3⍺ or GSK3β knockdown in MDA-MB-231 and MDA-MB-468 cells. CRISPR knockdown of GSK3⍺ or GSK3β in MDA-MB-231 (A,B,C,D) and MDA-MB-468 (E, F,G,H) cells were validated using western blotting. Immediately following transfection, some cells were extracted and used for Western blot analysis of GSK3⍺ or GSK3β. Representative blots are presented on the top and mean densitometric values are presented as ratio of β-actin in the histograms. Data are mean ± SEM values of three independent experiments. ** p < 0.01, *** p < 0.001. Individual blots and protein markers are shown in B, D, F and H.
